# Screening of protonstatin-1 (PS-1) analogs for improved inhibitors of plant plasma membrane H^+^-ATPase activity

**DOI:** 10.3389/fpls.2022.973471

**Published:** 2022-10-12

**Authors:** Yongqing Yang, Xiaohui Liu, Xin Wang, Wanjia Lv, Xiao Liu, Liang Ma, Haiqi Fu, Shu Song, Xiaoguang Lei

**Affiliations:** ^1^ College of Biological Sciences, China Agricultural University, Beijing, China; ^2^ Beijing National Laboratory for Molecular Sciences, Key Laboratory of Bioorganic Chemistry and Molecular Engineering of Ministry of Education, Department of Chemical Biology, College of Chemistry and Molecular Engineering, Synthetic and Functional Biomolecules Center, and Peking-Tsinghua Center for Life Sciences, Peking University, Beijing, China

**Keywords:** arabidopsis, plasma membrane H^+^-ATPase, inhibitor, auxin transport, PS-2

## Abstract

We previously identified protonstatin-1 (PS-1) as a selective inhibitor of plasma membrane H^+^-ATPase (PM H^+^-ATPase) activity and used it as a tool to validate the chemiosmotic model for polar auxin transport. Here, to obtain compounds with higher affinity than PS-1 for PM H^+^-ATPase, we synthesized 34 PS-1 analogs and examined their ability to inhibit PM H^+^-ATPase activity. The 34 analogs showed varying inhibitory effects on the activity of this enzyme. The strongest effect was observed for the small molecule PS-2, which was approximately five times stronger than PS-1. Compared to PS-1, PS-2 was also a stronger inhibitor of auxin uptake as well as acropetal and basipetal polar auxin transport in *Arabidopsis thaliana* seedlings. Because PS-2 is a more potent inhibitor of PM H^+^-ATPase than PS-1, we believe that this compound could be used as a tool to study the functions of this key plant enzyme.

## Introduction

Plasma membrane (PM) H^+^-ATPases are major proton transporters in plants and fungi (Palmgren, 2001; Yang and Guo, 2018a; Yang et al., 2022); they transport protons from the cytoplasm across the plasma membrane to the apoplast using energy generated by the hydrolysis of adenosine triphosphate (ATP). This creates a proton and chemical potential gradient across the plasma membrane (Falhof et al., 2016; Yang and Guo, 2018b; Chen et al., 2021), providing a driving force for the transport of other substances including ions, organic compounds, and so on across the plasma membrane (Falhof et al., 2016; Han et al., 2017; Yang and Guo, 2018b; Yang et al., 2021) while maintaining pH homeostasis in the cytoplasm and apoplast (Palmgren, 2001; Yang and Guo, 2018a; Havshøi and Fuglsang, 2022). The transport activity of numerous plasma membrane carrier proteins and channel proteins depends on the proton gradient and the potential gradient established by PM H^+^-ATPase (Yang et al., 2010; Yang et al., 2019a; Yang et al., 2019b; Havshøi and Fuglsang, 2022). PM H^+^-ATPase plays crucial roles in plant growth, development, and stress responses and is therefore referred to as the “master enzyme” in plants (Palmgren, 2001; Havshøi and Fuglsang, 2022).

Plants regulate many cellular processes by altering the activity of PM H^+^-ATPase. Therefore, understanding the mechanisms that regulate this pump is important for elucidating the physiological activities of plants. PM H^+^-ATPases in plants are encoded by a multigene family. For example, there are 11 homologous PM H^+^-ATPase genes (*AHAs*) in *Arabidopsis thaliana*, 10 in rice, 9 in tobacco, and 7 in tomato (Houlné and Boutry, 1994; Mito et al., 1996; Oufattole et al., 2000; Sperandio et al., 2011; Havshøi and Fuglsang, 2022). Because of functional redundancy, it is difficult to decipher the functions of the individual members of the PM H^+^-ATPase family. For example, in Arabidopsis, single PM H^+^-ATPase mutants often have no distinct phenotype, and *aha1 aha2* double mutants are embryonic lethal (Baxter et al., 2003; Gaxiola et al., 2007).

As an alternative to genetic approaches, the use of specific PM H^+^-ATPase inhibitors is an effective way to analyze the biological functions of PM H^+^-ATPases in plants. We previously identified protonstatin-1 (PS-1, (Z)-5-(furan-2-ylmethylene)-2-thioxothiazolidin-4-one), a selective inhibitor of PM H^+^-ATPase activity, from a small molecule library. PS-1 binds to the central loop of PM H^+^-ATPase and inhibits its activity (Yang et al., 2022).

The plant hormone auxin (indole-3-acetic acid [IAA]) exhibits polar transport from cell to cell (Muday and DeLong, 2001; Yang et al., 2006; Anfang and Shani, 2021; Yu et al., 2022). In the 1970s, the chemiosmotic model was proposed to explain the polar transport of auxin. According to this model, PM H^+^-ATPase plays a key role in regulating auxin transport (Rubery and Sheldrake, 1974; Went, 1974; Raven, 1975), which is mediated by diffusion and carriers, as supported by genetic analysis. To date, many auxin influx and auxin efflux transporters have been identified in plants (Zažímalová et al., 2010; Xi et al., 2019; Casanova-Sáez et al., 2021). For example, the model plant Arabidopsis contains four auxin influx transporters, AUX1, LAX1, LAX2, and LAX3, with AUX1 playing a major role in this process (Parry et al., 2001; Perrot-Rechenmann and Bennett, 2001; Kramer and Bennett, 2006). IAA is transported into cells through these transporters (Yang et al., 2006; Kerr and Bennett, 2007). Central to the chemiosmotic model is the dependence of IAA entry on the H^+^ gradient (or PM H^+^-ATPase), which is required for the diffusion and active transport of IAA. The transmembrane protein kinase TMK phosphorylates the PM H^+^-ATPase which is required for H^+^-ATPase activation and auxin-induced cell wall acidification (Li et al., 2021; Lin et al., 2021). Treatment of seedlings with PS-1 blocks auxin transport and inhibits auxin-controlled growth (Yang et al., 2022). Therefore, PS-1 was successfully used to validate the chemiosmotic model,

The affinity of PS-1 for the PM H^+^-ATPase proton pump is not very high, and the half-maximal inhibitory concentration is 3.9 μM. Therefore, in the current study, we sought out compounds with increased affinity for PM H^+^-ATPase by synthesizing 34 PS-1 analogs with modified functional groups and testing their effects on PM H^+^-ATPase activity. The PS-1 analogs showed varying inhibitory effects. PS-2 had the strongest inhibitory effect on PM H^+^-ATPase activity and was approximately 5-fold stronger than PS-1. PS-2 also inhibited polar auxin transport in Arabidopsis seedlings more strongly than PS-1. These findings suggest that PS-2 represents a more effective tool than PS-1 for studying the functions of PM H^+^-ATPases in plants.

## Results

### Design and synthesis of PS-1 analogs

**Figure 1:**
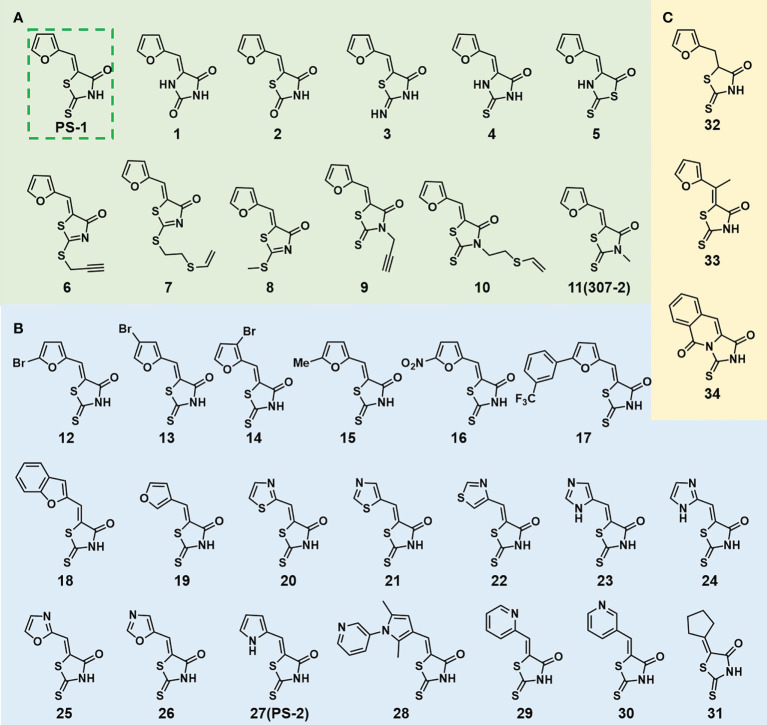
Synthesis of PS-1 analogs with altered functional groups. **(A)** PS-1 analogues with modifications on the rhodanine ring. **(B)** PS-1 analogs with modifications on the furan ring. **(C)** Other PS-1 analogs.

In order to explore the structure-activity relationships of the PS1 analogs, we divided PS-1 into two parts, the furan ring and the rhodanine ring, which are connected by an exocyclic double bond. Modification of PS-1 began with the replacement of the rhodanine ring with various five-membered heterocycle rings (1-5) (**Figure 1A**). We also introduced a series of chemical groups, including a propargyl group, a methyl group, or a vinyl thioethyl group, into the rhodanine ring at either the S (6-8) or N atom (9-11) (**Figure 1A**). In addition, we generated PS-1 derivatives with different substitutions on the furan ring (12-19) (**Figure 1B**) or PS-1 derivatives in which we replaced the furan ring with various aromatic or non-aromatic rings (20-31) (**Figure 1B**). We also reduced the double bond of PS-1 to a single bond (32) (**Figure 1C**) or introduced a methyl group to add steric hindrance (33) (**Figure 1C**). Finally, we generated a more constrained compound that was more rigid than PS-1 (34) (**Figure 1C**). In all, we generated 34 analogs to explore the structure-activity relationship and to identify more potent PM H^+^-ATPase inhibitors.

### Verification of the inhibition of PM H^+^-ATPase activity by PS-1 analogs in isolated Arabidopsis vesicles

**Figure 2:**
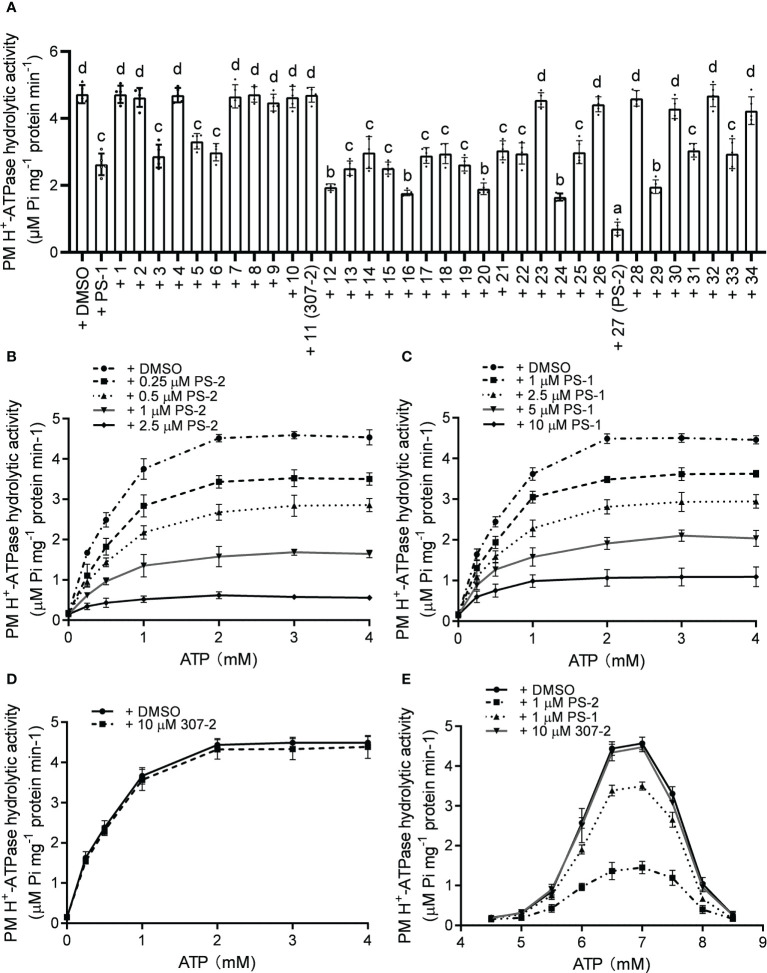
Effects of PS-2 on PM H^+^-ATPase hydrolytic activity. **(A)** Effects of PS-1 analogs on PM H^+^-ATPase hydrolytic activity. Plasma membrane vesicles were isolated from Arabidopsis Col-0 seedlings that were treated with 250 mM NaCl for 3 d before harvest. The inhibitory effects of 10 μM PS-1 analogs or 0.1% (v/v) DMSO on PM H^+^-ATPase hydrolytic activity were assessed. Data are means ± SD of five replicates. **(B)** Kinetic analysis of PM H^+^-ATPases inhibition by PS-2. **(C)** Kinetic analysis of PM H^+^-ATPases inhibition by PS-1. **(D)** Kinetic analysis of PM H^+^-ATPases inhibition by 307-2. **(E)** PM H^+^-ATPase activity assayed between pH 4.5 and pH 8.5 in the presence or absence of PS-2. Different lowercase letters indicate significant differences (P ≤ 0.05), as determined by one-way ANOVA.

To verify the inhibitory effects of the PS-1 analogs on PM H^+^-ATPase activity, we isolated plasma membrane vesicles from Arabidopsis seedlings and measured the hydrolysis activity of PM H^+^-ATPase following treatment with these analogs. The inhibitory effects of the PS-1 analogs on PM H^+^-ATPase activity in Arabidopsis plasma membrane vesicles were consistent with the inhibitory effects measured in the RS72 yeast system (**Figure 2A**, **Supplementary Figure 1**). The inhibitory effect of analog PS-2 was the strongest. Compared to the DMSO control, treatment with 5 μM PS-2 inhibited approximately 85% of PM H^+^-ATPase activity, while the same concentration of PS-1 only inhibited ~44% of this activity (**Figure 2A**). To characterize the mode of H^+^-ATPase inhibition by PS-2, we measured enzyme kinetics in the presence of various concentrations of PS-2 (0.25, 0.5, 1 and 2.5 μM), PS-1 (1, 2.5, 5 and 10 μM) or 307-2 (10 μM) with increasing concentrations of the substrate ATP (0.25-4 mM). Total PM H^+^-ATPase hydrolysis activity is shown in **Figures 2B–D**, and the initial plots indicated non-competitive inhibition, so the curves were fitted by appropriate nonlinear regression. Compared to control DMSO, Km values for ATP remained relatively constant regardless of the addition of PS-2, PS-1 or 307-2 (**Supplementary Tables 1–3**). On the other hand, Vmax values decreased due to increasing concentration of PS-2, PS-1 or 307-2 (**Supplementary Tables 1–3**). These kinetic data suggest that PS-2 behaves as a noncompetitive inhibitor of ATP. We then determined the effect of PS-2 on the optimum pH for the hydrolytic activity of H^+^-ATPase. The results showed that the maximum activity of PM H^+^-ATPase was close to pH 6.5 in the presence or absence of PS-2 (**Figure 2E**).

### Testing the ability of PS-1 analogs to inhibit PM H^+^-ATPase activity in yeast strain RS72 cells expressing Arabidopsis AHA2

**Figure 3:**
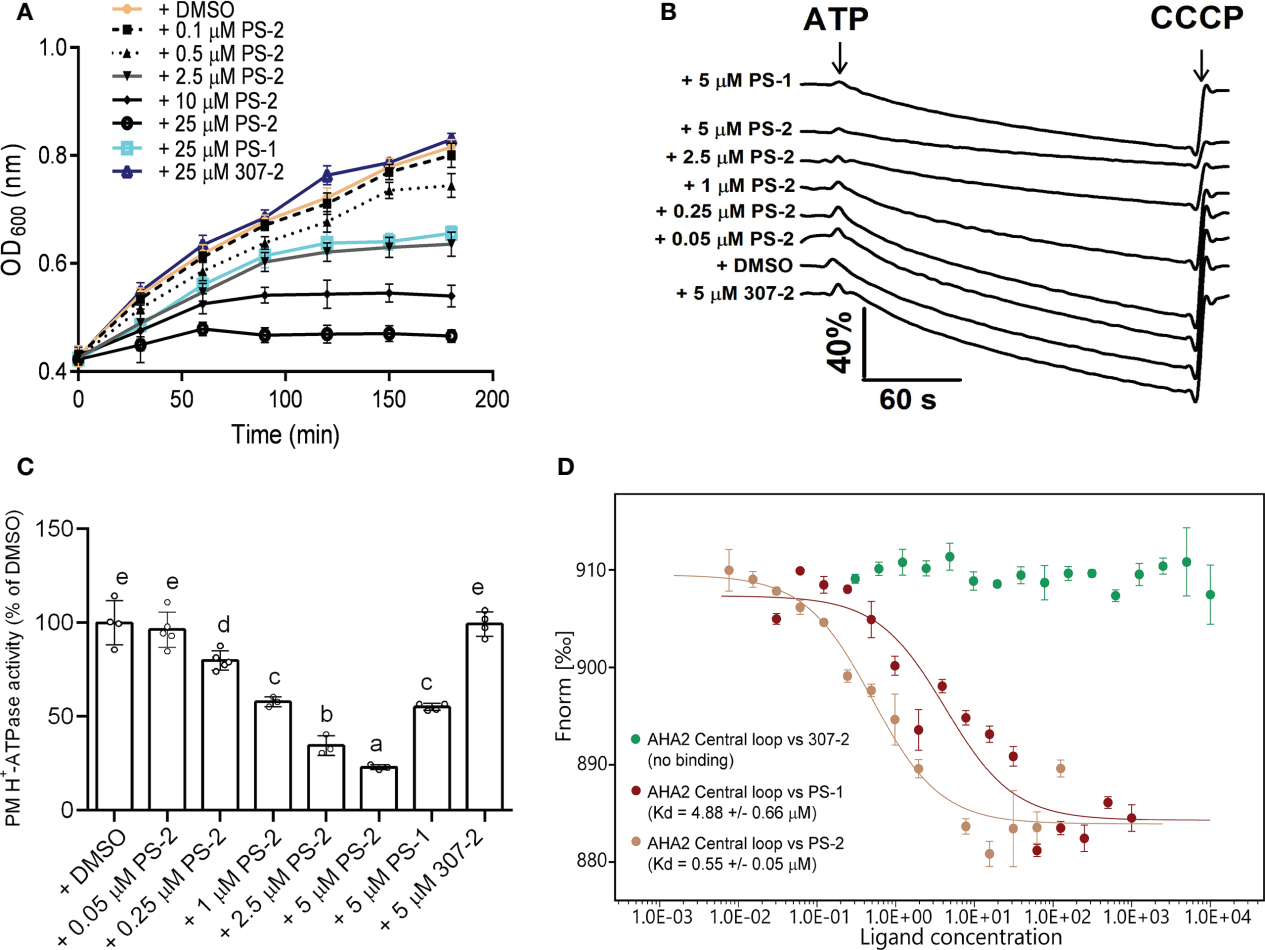
Effects of PS-2 on PM H^+^-ATPase proton pumping activity. **(A)** Effect of PS-2 on the growth of yeast strain RS72-AHA2. Yeast cells were treated with 0.05% (v/v) DMSO (vehicle control), 25 μM PS-1(positive control), 25 μM 307-2 (negative control) or with various PS-2 concentrations in 96-well plates. Yeast growth was followed as OD_600 nm_. Data are means ± standard deviation (SD) from at least ten replicates. **(B)** Effect of PS-2 on PM H^+^-ATPase proton pumping activity in PM vesicles isolated from Arabidopsis Col-0 seedlings that were treated with 250 mM NaCl for 3 d before harvest. PM H^+^-ATPase proton pumping activity was initiated by the addition of 3 mM ATP (final concentration) and collapsed by the addition of 10 μM (final concentration) carbonyl cyanide m-chlorophenylhydrazone (CCCP). Data represents one representative experiment from at least three replicates. **(C)** PM H^+^-ATPase proton pumping activity in vesicles as a function of PS-2 concentration. Inhibitor-dependent changes of values of proton pumping activity during the 60 s were normalized to DMSO control, which was set to 100%. Data represent means ± SD from at least three replicates. Each replicate was performed using independent membrane preparations. **(D)** Binding affinity of PS-2, PS-1(positive control), 307-2 (negative control), to recombinant His-AHA2 central loop, as determined by MST. The difference in normalized fluorescence Fnorm [‰] is shown. Kd values are means ± SD (n = 3). Different lowercase letters indicate significant differences (P ≤ 0.05), as determined by one-way ANOVA.

To identify compounds that are stronger PM H^+^-ATPase inhibitors than PS-1, we screened the 34 PS-1 analogs by observing their inhibitory effects on the growth of RS72 yeast cells expressing the Arabidopsis PM H^+^-ATPase AHA2. In RS72 yeast cells, the expression of *PMA1*, yeast’s endogenous PM H^+^-ATPase gene, is controlled by the galactokinase 1 (*GAL1*) promoter. Although yeast contains two PM H^+^-ATPases, PMA1 and PMA2, unlike PMA1, PMA2 is expressed at low levels and is not required for yeast growth (Ghislain and Goffeau, 1991). RS72 yeast cannot survive on medium containing glucose as the sole carbon source, but heterologous expression of the Arabidopsis *AHA2* gene under the control of the yeast *PMA1* promoter (Villalba et al., 1992; Regenberg et al., 1995) rescues this growth defect (Regenberg et al., 1995; Kjellerup et al., 2017). Inhibition of AHA2 activity in yeast strain RS72-AHA2 using PS-1 resulted in decreased yeast cell growth. Therefore, we performed a phenotypic screen of PS-1 analogs based on the growth of yeast RS72-AHA2 cells to identify PM H^+^-ATPase inhibitors that are more potent than PS-1. In an initial screen (**Supplementary Figure 1**), we identified several analogs that were more potent than PS-1 in inhibiting the growth of RS72 yeast, with PS-2 being the most potent. Furthermore, we measured a dose-dependent inhibition of yeast strain RS72-AHA2 by PS-2 (0.1-25 μM) and proton pumping activity (0.05-5 μM in isolated plasma membrane vesicles from Arabidopsis) (**Figures 3A–C**). The results of plasma membrane vesicles showed that PS-2 was about 5 times more effective than PS-1, and the inhibitory effect of 1 μM PS-2 on PM H^+^-ATPase was comparable to that of 5 μM PS-1 (**Figure 3C**). Besides, we also measured the binding constant between PS-2 and the AHA2 central loop using microscale thermophoresis (MST). A 10-fold boost in affinity between the AHA2 central loop and PS-2 interaction was observed (K_d_=0.55 ± 0.05) relative to the AHA2 central loop-PS-1 (K_d_=4.88 ± 0.66 μM), while no measurable binding was detected between the 307-2 and AHA2 central loop (**Figure 3D**).

### Effects of PS-1 analogs on Arabidopsis seedling growth

**Figure 4:**
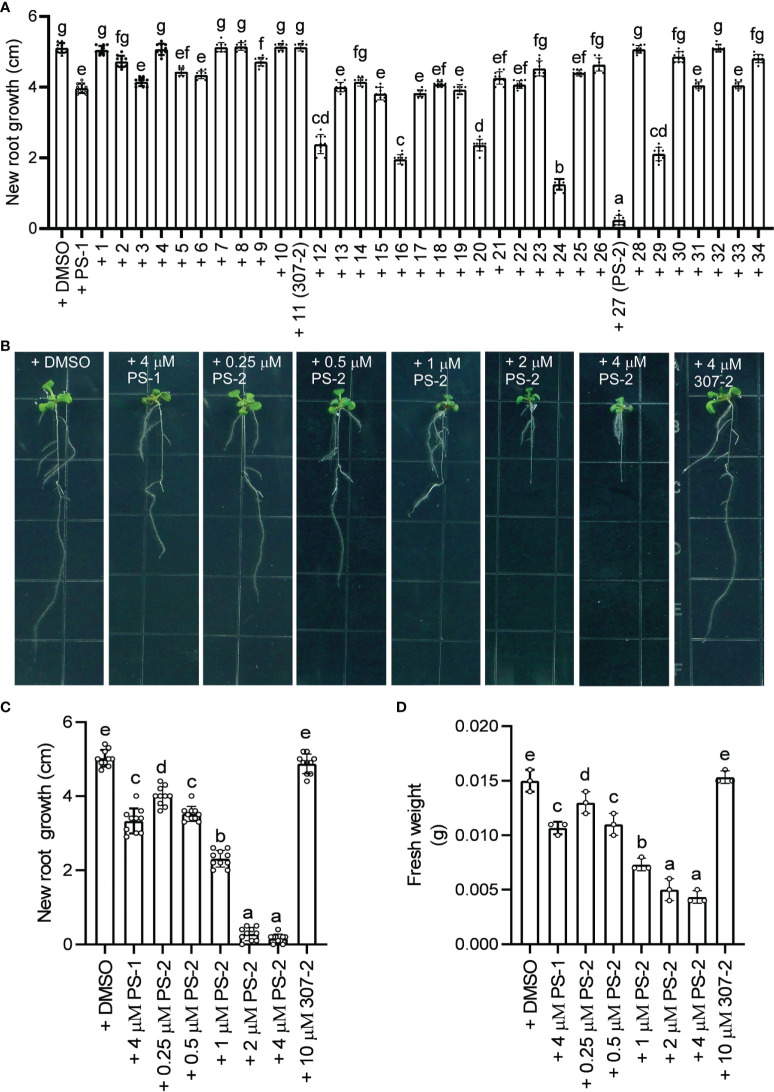
Effects of PS-2 on Arabidopsis seedling growth. **(A)** New growth of primary roots shown in **Supplementary Figure 2** during the course of the experiment. Data are means ± SD of 10 plants. Different lowercase letters indicate significant differences (*P* ≤ 0.05), as determined by one-way ANOVA. **(B)** Representative photographs of PS-2-treated seedlings. Five-day-old seedlings grown on MS medium, pH 5.8, were transferred to plates containing indicated PS-2 or 0.05% (v/v) DMSO (vehicle control), 4 μM PS-1(positive control), 4 μM 307-2 (negative control) and incubated for 7 d at 22°C for 7 d prior to photography. **(C)** Lengths of new growth primary roots in **Figure 3B** were measured 7 days after transfer. Data represent mean values ± SD (n = 10 replicates). **(D)** Analysis of fresh weight for seedlings in **Figure 3B**. Error bars represent SD (n = 3, 3 plates, 4 seedlings on each plate are weighed together) from the measurements using the same preparation.

We previously demonstrated that PS-1 inhibits PM H^+^-ATPase activity and Arabidopsis seedling growth (Yang et al., 2022). In our screen of 34 PS-1 analogs, we identified several with stronger inhibitory effects than PS-1 on PM H^+^-ATPase activity (**Supplementary Figure 1**, **Figure 2A**). To further verify the inhibitory effects of these analogs, we observed their effects (at 2.5 μM) on Arabidopsis seedling growth. As expected, the ability of the PS-1 analogs to inhibit PM H^+^-ATPase was consistent with their ability to inhibit Arabidopsis root growth: compounds that strongly inhibited PM H^+^-ATPase activity also strongly inhibited Arabidopsis root growth (**Supplementary Figure 2**), with analog PS-2 having the strongest inhibitory effect (**Supplementary Figure 2**, **Figure 4A**). In addition, we observed a dose-dependent inhibition of Arabidopsis seedling growth by PS-2 (0.5-4 μM). Specifically, PS-2 blocked primary root growth and fresh weight in a dose-dependent manner, and the inhibitory effect of 0.5 μM PS-2 was comparable to that of 4 μM PS-1 (**Figures 4B–D**).

### The effect of PS-1 analog PS-2 on polar auxin transport

**Figure 5:**
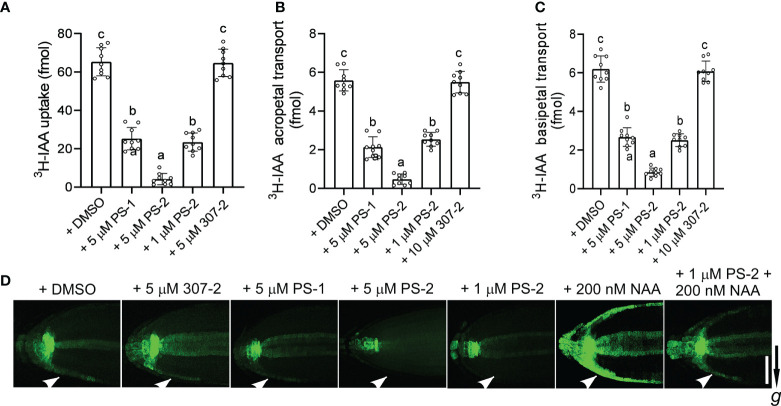
Effects of PS-1 analogs on polar auxin transport. **(A)** Uptake of ^3^H-labeled IAA in root tips of Col-0 seedlings. Ten apical hypocotyl segments (each 5 mm long) were placed in 25 μL of buffer (5 nM ^3^H-labeled IAA, 1 μM IAA, 1% [w/v] sucrose, and 5 mM sodium phosphate, pH 6.0) containing the indicated concentration of PS-1 analog (0.1% [v/v] DMSO as a control). After shaking for 2 h (100 rpm/min) at room temperature, the amount of ^3^H-IAA in the segments was measured. Data are means ± SD of 9 replicates. **(B, C)** Effects of PS-1 analogs on acropetal and basipetal transport of ^3^H-labeled IAA in the roots of Arabidopsis Col-0 seedlings. Seven-day-old seedlings were placed on square agar plates containing the indicated concentration of PS-1 analog (0.1% [v/v] DMSO as a control). Agar pieces containing 100 nM ^3^H-labeled IAA were placed below the shoot–root junction for 12 h (B, acropetal transport) or at the root tip for 6 h (C, basipetal transport). The amount of ^3^H-labeled IAA in segments 5 mm from root tip **(B)** or from 10 to 15 mm **(C)** above the root apex was determined. Data are means ± SD of 9 biological replicates. Different lowercase letters indicate significant differences (*P* ≤ 0.05), as determined by one-way ANOVA. **(D)** Effects of PS-1 on asymmetric auxin accumulation in response to gravity. Seven-day-old *DR5:GFP* transgenic seedlings were placed on square agar plates containing the indicated concentration of PS-1 analog (or 0.1% [v/v] DMSO as a control) and rotated 90° for 4 h prior to imaging. One representative image is shown out of 10 seedlings imaged in the experiments. The arrow indicates the direction of gravity. Bar = 100 μm.

We previously demonstrated that PS-1 inhibits PM H^+^-ATPase activity and inhibits polar auxin transport in Arabidopsis roots (Yang et al., 2022). We observed that the PS-1 analog PS-2 had a significantly stronger inhibitory effect than PS-1 on PM H^+^-ATPase activity. To compare the effects of PS-2 and PS-1 on polar auxin transport, we examined ^3^H-IAA transport and the expression of the auxin-responsive reporter gene *DR5-GFP* in Arabidopsis roots in the presence of these compounds. Compared to the DMSO control, the uptake of ^3^H-IAA was significantly reduced in the presence of 5 μM PS-1 or PS-2 (**Figure 5A**). As expected, the inhibitory effect of PS-2 was significantly stronger than that of PS-1. The inhibitory effect of 1 μM PS-2 was similar to that of 5 μM PS-1, whereas 5 μM 307-2 (a PS-1 analog that did not significantly inhibit PM H^+^-ATPase activity) had no apparent effect on auxin uptake (**Figure 5A**). We also examined auxin transport in Arabidopsis roots treated with PS-2 or PS-1 by measuring their acropetal and basipetal polar transport of ^3^H-IAA. Treatment with 5 μM PS-2 or PS-1 significantly reduced both acropetal and basipetal ^3^H-IAA polar transport in roots, but the inhibitory effect of PS-2 was significantly stronger than that of PS-1 (**Figures 5B, C**). By contrast, 307-2 had no effect on polar ^3^H-IAA transport (**Figures 5B, C**).

Finally, we measured the effects of PS-2 and PS-1 on the redistribution of auxin in response to gravity by examining the expression of the auxin reporter DR5-GFP in Arabidopsis roots. As expected, untreated roots expressed DR5-GFP asymmetrically under gravistimulation, with enhanced DR5-GFP expression in the outermost cell layer on the underside of the root tip (**Figure 5D**). In seedlings treated with PS-2 or PS-1, this localized enhancement of DR5-GFP expression was attenuated, and the inhibitory effect of PS-2 was significantly stronger than that of PS-1 (**Figure 5D**). By contrast, 307-2 had no effect on the asymmetric auxin accumulation in response to gravity (**Figure 4D**). In roots exposed to 200 nM NAA (an auxin) for 4 hours, DR5-GFP was strongly expressed in the outer layer of both sides of the root tip. However, 1 μM PS-2 reduced the DR5-GFP signal induced by NAA treatment (**Figure 5D**). Taken together, these results indicate that PS-2 is a strong inhibitor of polar auxin transport.

## Discussion

We synthesized 34 analogs of the PM H^+^-ATPase inhibitor PS-1 by systematically altering its functional groups and then screened these analogs for enhanced inhibitory activity. Structure-activity analysis showed that replacement of the rhodanine ring or modification of it compromised or totally abolished the inhibitory effects on both the yeast growth and the Arabidopsis seedling growth, suggesting the rhodanine is critical for the binding to PM H^+^-ATPase. Modification on the furan ring, however, is tolerable, and the introduction of an electron-withdrawing group (Bromo or nitro group) at the 5- position improved the inhibitory effect (compounds 12 and 16), while replacement of the furan ring with nitrogen-containing five membrane (compounds 20, 24, and 27), especially those with a hydrogen bond donor (24 and 27), markedly increased the potency. The analog 27, named PS-2, had the strongest inhibitory effects on PM H^+^-ATPase activity and polar auxin transport, and was clearly a stronger inhibitor than PS-1. Because it has a better inhibitory effect than PS-1, PS-2 should be a useful tool to study the biological functions of PM H^+^-ATPase.

We previously tested the effect of PM H^+^-ATPase activity on polar auxin transport by treating the roots of Arabidopsis seedlings with selective inhibitor PS-1 of PM H^+^-ATPase (Yang et al., 2022). The results supported the chemiosmotic model that PM H^+^-ATPase mediates polar auxin transport. In the current study, we identified PS-2, a PS-1 analog with a stronger inhibitory effect on PM H^+^-ATPase than PS-1. PS-2 inhibited the polar transport of auxin, further verifying the chemiosmotic model. PS-2 represents a potential tool to further explore the physiological roles of PM H^+^-ATPases.

## Materials and methods

### Plant materials

*Arabidopsis thaliana* Col-0 seeds were surface sterilized, germinated on Murashige and Skoog (MS) medium plus 0.6% (w/v) Phytagel (Sigma, lot # WXBC6825V) and 2.5% (w/v) sucrose, incubated in the dark at 4°C for 48 h, and vertically grown in a controlled growth chamber under a 16-h-light/8-h-dark cycle (22°C) for 5 d. The seedlings were transferred to control plates (containing 0.1% [v/v] DMSO) or plates containing 2.5 μM PS-1 or PS-1 analogs (in 0.1% [v/v] DMSO) and incubated for 7 d under a 16-h-light/8-h-dark cycle (22°C).

### Screening of PS-1 analogs and PS-2 dose-dependent inhibition assay

We screened PS-I analogs in yeast strain RS72 expressing Arabidopsis AHA2, which was constructed as previously reported (Regenberg et al., 1995). Yeast cells were pre-cultured in flasks to an OD_600_ of 0.5 and diluted at a ratio of 1:5 or 1:25 in fresh medium. A 4 μL aliquot of yeast cells was spotted onto solid medium containing DMSO as a control (0.1% [v/v]) or 10 μM PS-1 or PS-1 analog and photographed after 3 days of incubation at 28°C.

In the PS-2 dose-dependent inhibition assay against yeast strain RS72, yeast cells were pre-cultured in flasks until OD_600 nm_ reached approximately 0.4. Yeast cells were then aliquoted into 96-well plates, receiving 200 μL of culture per well in the presence of the DMSO control (0.1% [v/v]) or various compounds (0.1 to 25 μM of PS-2, 25 μM of PS-1 or 25 μM of 307-2). Yeast growth (OD_600_ nm) was monitored using a microplate reader (Bio-Tek) after shaking at 28°C at 220 rpm/min for 3 h.

### Assay of PM H^+^-ATPase hydrolytic activity and proton pumping activity

Plasma membrane-enriched vesicles were isolated from 3-week-old Arabidopsis seedlings using two-phase partitioning (Qiu et al., 2002; Yang et al., 2010). PM H^+^-ATPase hydrolytic activity was measured as described previously (Qiu et al., 2004) in the presence of the DMSO control (0.1% [v/v]) or 10 μM PS-1 or PS-1 analog. The proton pumping activity was measured in the presence of the DMSO control (0.1% [v/v]) or various compounds (0.05 to 5 μM of PS-2, 5 μM of PS-1 or 5 μM of 307-2) as described previously (Qiu et al., 2002).

### Microscale thermophoresis assay

The interaction between the AHA2 central cyclic peptide and PS-2 was detected by MST analysis using the NanoTemper Monolith NT.115 instrument as previously described (Wienken et al., 2010). Recombinant AHA2 central loop peptide was labeled with the Monolith NTTM Protein Labeling Kit RED (http://www.nanotemper-technologies.com/). The concentration of various compounds (PS-2, PS-1 or 307-2) ranged from 0.015 to 1000 μM. The labeled AHA2 central loop peptide was used in PBS buffer (10 nM in phosphate, pH 7.6) containing 0.05% (v/v) Tween 20 (PBST). The labeled peptide and compound mixture was incubated in PBST buffer for 5 min in the dark and then transferred to standard treated capillaries for measurement.

### IAA transport assay

Auxin uptake in Arabidopsis roots was measured as previously described (Rahman et al., 2001). Assays for acropetal and basipetal transport of auxin in Arabidopsis roots were performed as described previously (Lewis and Muday, 2009).

### Synthetic schemes, methods, and characterizations

^1^H NMR spectra were performed on a Varian or Bruker 400 MHz spectrometer at ambient temperature with CDCl_3_ or DMSO-d_6_ as the solvent unless otherwise addressed. ^13^C NMR spectra were recorded on a Varian or Bruker 100 MHz spectrometer at ambient temperature (with complete proton decoupling). Chemical shifts are reported in parts per million relative to DMSO-d_6_ (^1^H, δ 2.50; ^13^C, δ 39.50) or chloroform (^1^H, δ 7.26; ^13^C, δ 77.00). Data for ^1^H NMR are reported as follows: chemical shift, multiplicity (s = singlet, d = doublet, m = multiplet), coupling constants and integration. High-resolution mass spectra were obtained at Peking University Mass Spectrometry Laboratory using Bruker APEX Flash chromatography. All chemical reagents were used as supplied by Alfa Aesar Chemicals, Sigma-Aldrich, J&K, and Acros. EtOH was distilled from calcium hydride; tetrahydrofuran (THF) was distilled from sodium/benzophenone prior to use. Compound 1-2, 4, 6, 8-9, 11, 15-18, 25, 28-31, 33-34 are the same as literature reported.

*General procedure*: To a solution of the aldehyde (1 equiv) and the heterocycle compound (1 equiv) in acetic acid was added ammonium acetate (2 equiv). The mixture was stirred at reflux under an argon atmosphere. After 4 h, the reaction mixture was cooled to room temperature and the precipitates were collected by filtration and washed with distilled water. The title compound was recrystallized in ethanol.


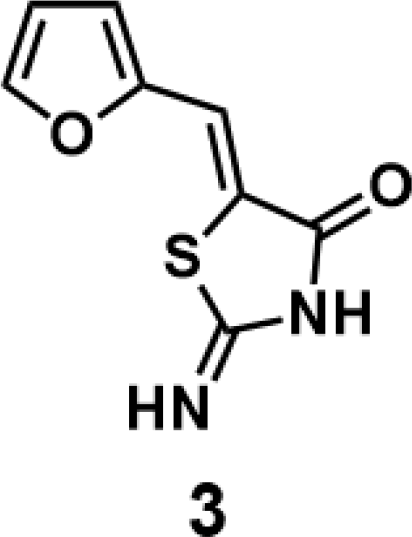
 (*Z*)-5-(furan-2-ylmethylene)-2-iminothiazolidin-4-one(3): prepared according to general procedure with furfural and 2-iminothiazolidin-4-one to yield compound 3 (128.4 mg, 0.662 mmol, 77%). ^1^H NMR (400 MHz DMSO-d_6_): δ 6.69 (dd, *J* = 3.2 Hz, 1.6 Hz, 1H), 6.93 (d, *J* =3.6 Hz, 1H), 7.39 (s, 1H), 7.95 (d, *J* = 1.6 Hz, 1H), 9.05 (br, 1H), 9.32 (br, 1H); ^13^C NMR (100 MHz DMSO-d_6_): δ 113.2, 115.8, 116.1, 126.9, 146.1, 149.7, 176.1, 180.1. HRMS (ESI): [M + H]^+^ calculated for C_8_H_7_N_2_O_2_S: 195.0223, found: 195.0224.


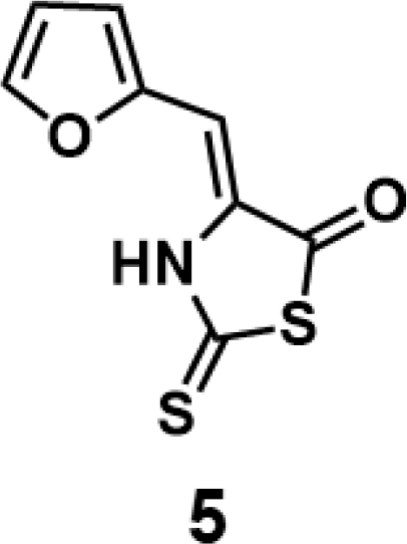
 (*Z*)-4-(furan-2-ylmethylene)-2-thioxothiazolidin-5-one(5): prepared according to general procedure with furfural and 2-thioxothiazolidin-5-one to yield compound 5 (13.4 mg, 0.636 mmol, 63%). ^1^H NMR (400 MHz DMSO-d_6_): δ 6.65(s, 1H), 6.75 (dd, *J* = 3.6 Hz, 2 Hz, 1H), 7.29 (d, *J* = 3.6 Hz, 1H), 8.01 (d, *J* = 1.6 Hz, 1H);^13^C NMR (100 MHz DMSO-d_6_): δ102.3, 113.8, 119.4, 131.0, 147.5, 148.4, 187.7, 188.2; HRMS (ESI): [M + H]^+^ calculated for C_8_H_6_NO_2_S_2_: 211.9835, found: 211.9836.


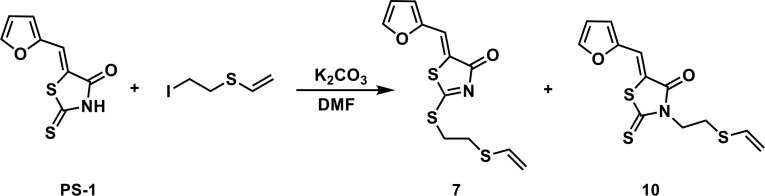
 To a solution of PS-1(51 mg, 0.241 mmol) and (2-iodoethyl)(vinyl)sulfane (77.4 mg, 0.362 mmol) in 2 mL of DMF was added K_2_CO_3_ (50 mg, 0.362 mmol). The mixture was stirred under the atmosphere of argon at room temperature overnight. The mixture was then concentrated in vacuo and the residue was purified by thin-layer chromatography to afford compounds 7 (53.9 mg, 0.181 mmol, 75.2%) and 10 (16.7 mg, 0.0562 mmol, 23.3%).


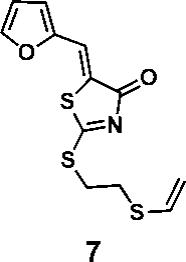
 (*Z*)-5-(furan-2-ylmethylene)-2-((2-(vinylthio)ethyl)thio)thiazol-4(5H)-one (7): ^1^H NMR (400 MHz CDCl_3_): δ 3.12-3.17 (m, 2H), 3.58-3.63(m, 2H), 5.29(d, *J* = 16.8 Hz, 1H), 5.31(d, *J* = 10 Hz, 1H), 6.35 (dd, *J* = 16.8 Hz, 10 Hz, 1H), 6.58 (dd, *J* = 3.2 Hz, 1.8 Hz, 1H), 6.82 (d, *J*=3.6 Hz 1H), 7.61 (s, 1H), 7.66 (m, 1H); HRMS (ESI): [M + H]^+^ calculated for C_12_H_12_NO_2_S_3_: 298.0025, found: 298.0029.


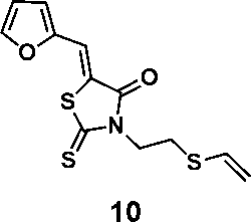
 (*Z*)-5-(furan-2-ylmethylene)-2-thioxo-3-(2-(vinylthio)ethyl)thiazolidin-4-one (10): ^1^H NMR (400 MHz CDCl_3_): δ 3.01 (t, *J* = 7.4 Hz, 2H), 4.34(t, *J* = 7.4 Hz, 2H), 5.29(d, *J* = 10 Hz, 1H), 5.35 (d, *J* = 16.8 Hz, 1H), 6.36 (dd, *J* = 16.8 Hz, 10 Hz, 1H), 6.60 (m, 1H), 6.85 (d, *J*=3.6 Hz 1H), 7.48 (s, 1H), 7.71 (s, 1H); ^13^C NMR (100 MHz CDCl_3_): δ 27.6, 43.2, 112.5, 113.6, 118.6, 118.9, 120.5, 130.6, 147.2, 150.0, 167.3, 194.3; HRMS (ESI): [M + H]^+^ calculated for C_12_H_12_NO_2_S_3_: 298.0025, found: 298.0027.


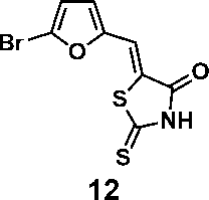
 (*Z*)-5-((5-bromofuran-2-yl)methylene)-2-thioxothiazolidin-4-one(12): prepared according to general procedure with 5-bromofuran-2-carbaldehyde and rhodanine to yield compound 12 (53 mg, 0.262 mmol, 64%). ^1^H NMR (400 MHz DMSO-d_6_): δ 6.91 (d, *J* = 4 Hz, 1H), 7.20 (d, *J* = 3.6 Hz, 1H), 7.42 (s, 1H), 13.75 (br, 1 H); ^13^C NMR (100 MHz DMSO-d_6_): δ 116.0, 116.3, 121.7, 123.2, 128.0, 151.5, 168.9,196.1; HRMS (ESI): [M + H]^+^ calculated for C_8_H_5_BrNO_2_S_2_: 289.8940, found: 289.8942.


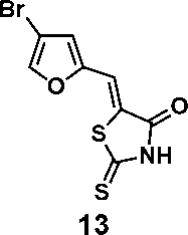
 (*Z*)-5-((4-bromofuran-2-yl)methylene)-2-thioxothiazolidin-4-one(13): prepared according to general procedure with 4-bromofuran-2-carbaldehyde and rhodanine to yield compound 12 (133 mg, 0.332 mmol, 81%). ^1^H NMR (400 MHz DMSO-d_6_): δ 7.31 (d, *J* = 2 Hz, 1H), 7.42 (s, 1H), 8.34 (s, 1H), 13.78 (br, 1 H); ^13^C NMR (100 MHz DMSO-d_6_): δ 102.5, 116.3, 120.8, 124.5, 146.0, 150.3, 168.9, 196.2, HRMS (ESI): [M + H]^+^ calculated for C_8_H_5_BrNO_2_S_2_: 289.8940, found: 289.8942.


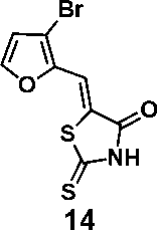
 (*Z*)-5-((3-bromofuran-2-yl)methylene)-2-thioxothiazolidin-4-one(14): prepared according to general procedure with 3-bromofuran-2-carbaldehyde and rhodanine to yield compound 12 (53.9 mg, 0.188 mmol, 59%). ^1^H NMR (400 MHz DMSO-d_6_): δ 7.06 (d, *J* = 2 Hz, 1H), 7.17 (s, 1H), 8.20 (d, *J*= 2 Hz, 1H), 12.92 (br, 1 H); ^13^C NMR (100 MHz DMSO-d_6_): δ 108.9, 112.9, 116.8, 124.9, 146.6, 148.9, 169.0,

196.1;HRMS (ESI): [M + H]^+^ calculated for C_8_H_4_BrNO_2_S_2_: 289.8940, found: 289.8943.


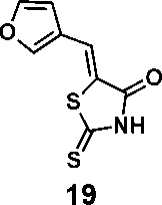
 (*Z*)-5-(furan-3-ylmethylene)-2-thioxothiazolidin-4-one(19): prepared according to general procedure with furan-3-carbaldehyde and rhodanine to yield compound 19 (58.4 mg, 0.277 mmol, 74%). ^1^H NMR (400 MHz DMSO-d_6_): δ 6.78 (d, *J* = 2 Hz, 1H), 7.58 (s, 1H), 7.91 (m, 1H), 8.32 (s, 1H), 13.75 (bs, 1 H); ^13^C NMR (100 MHz DMSO-d_6_): δ 109.4, 121.4, 123.4, 124.9, 146.5, 148.6, 169.5, 195.6; HRMS (ESI): [M + H]^+^ calculated for C_8_H_6_NO_2_S_2_: 211.9835, found: 211.9835.


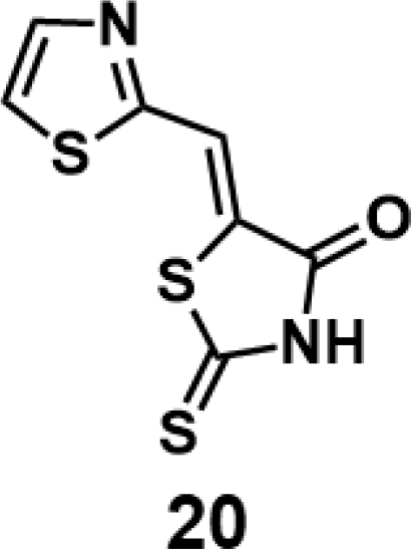
 (*Z*)-5-(thiazol-2-ylmethylene)-2-thioxothiazolidin-4-one (20): prepared according to general procedure with thiazole-2-carbaldehyde and rhodanine to yield compound 20 (98 mg, 0.429 mmol, 97%). ^1^H NMR (400 MHz DMSO-d_6_): δ 7.91 (s, 1H), 8.09 (d, *J* = 3.2 Hz, 1H), 8.21 (d, *J* = 3.2 Hz, 1H), 13.79 (br, 1H); ^13^C NMR (100 MHz DMSO-d_6_): δ 119.9, 125.4, 129.3, 145.4, 160.7, 168.9, 199.7; HRMS (ESI): [M + H]^+^ calculated for C_7_H_5_N_2_OS_3_: 228.9559, found: 228.9561.


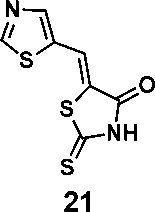
 (*Z*)-5-(thiazol-5-ylmethylene)-2-thioxothiazolidin-4-one(21): prepared according to general procedure with thiazole-5-carbaldehyde and rhodanine to yield compound 21 (83.8 mg, 0.367 mmol, 83%). ^1^H NMR (400 MHz DMSO-d_6_): δ 8.03 (m, 1H), 8.05 (m, 1H), 9.46 (s, 1H), 13.95 (br, 1H); ^13^C NMR (100 MHz DMSO-d_6_): δ 121.6, 126.9, 123.1, 149.4, 159.7, 168.6, 194.2; HRMS (ESI): [M + H]^+^ calculated for C_7_H_5_N_2_OS_3_: 228.9559, found: 228.9561.


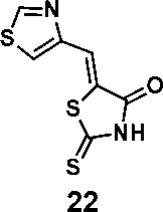
 (*Z*)-5-(thiazol-4-ylmethylene)-2-thioxothiazolidin-4-one(22): prepared according to general procedure with thiazole-4-carbaldehyde and rhodanine to yield compound 22 (84.8 mg, 0.371 mmol, 84%). ^1^H NMR (400 MHz DMSO-d_6_): δ 7.70 (s, 1H), 8.40 (m, 1H), 9.34 (d, *J* = 1.2 Hz, 1H), 13.63 (bs, 1H); ^13^C NMR (100 MHz DMSO-d_6_): δ 122.7, 127.0, 127.4, 150.1, 156.3, 169.4, 199.4; HRMS (ESI): [M + H]^+^ calculated for C_7_H_5_N_2_OS_3_: 228.9559, found: 228.9561.


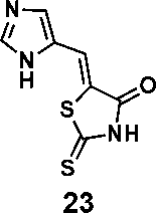
 (*Z*)-5-((1*H*-imidazol-5-yl)methylene)-2-thioxothiazolidin-4-one(23): prepared according to general procedure with 1*H*-imidazole-5-carbaldehyde and rhodanine to yield compound 23 (251.4 mg, 0.2444 mmol, 47%). ^1^H NMR (400 MHz DMSO-d_6_): δ 7.53 (s, 1H), 7.84 (d, 1H), 7.96 (s, 1H), 12.75 (br, 1H), 13.40 (br, 1H); ^13^C NMR (100 MHz DMSO-d_6_): δ 121.3, 124.1, 124.5, 135.7, 138.5, 169.4, 199.7. HRMS (ESI): [M + H]^+^ calculated for C_7_H_6_N_3_OS_2_: 211.9947, found: 211.9948.


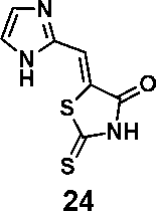
 (*Z*)-5-((1*H*-imidazol-2-yl)methylene)-2-thioxothiazolidin-4-one(24): prepared according to general procedure with 1*H*-imidazole-2-carbaldehyde and rhodanine to yield compound 24 (5.9 mg, 0.123 mmol, 23.7%). ^1^H NMR (400 MHz DMSO-d_6_): δ 7.32 (s, 1H), 7.35 (s, 1H), 7.50 (s, 1H), 12.93 (bs, 1H), 13.50 (bs, 1H); ^13^C NMR (100 MHz DMSO-d_6_): δ 116.7, 121.0, 125.6, 132.5, 142.0, 169.1, 199.7; HRMS (ESI): [M + H]^+^ calculated for C_7_H_6_N_3_OS_2_: 211.9947, found: 211.9948.


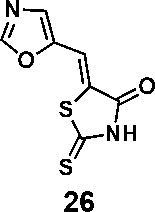
 (*Z*)-5-(oxazol-5-ylmethylene)-2-thioxothiazolidin-4-one (26): prepared according to general procedure with oxazole-5-carbaldehyde and rhodanine to yield compound 26 (69.8 mg, 0.33 mmol, 64%). ^1^H NMR (400 MHz DMSO-d^6^): δ 7.59 (s, 1H), 7.79 (s, 1H), 8.74 (s, 1H), 13.80 (br, 1H); ^13^C NMR (100 MHz DMSO-d^6^): δ 114.6, 126.0, 133.2, 146.8, 155.0, 168.8, 196.0; HRMS: [M + H]^+^ calculated for C_7_H_5_N_2_O_2_S_2_: 212.9787, found: 212.9789.


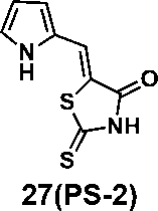
 (*Z*)-5-((1*H*-pyrrol-2-yl)methylene)-2-thioxothiazolidin-4-one(27): prepared according to general procedure with 1*H*-pyrrole-2-carbaldehyde and rhodanine to yield compound 27 (53 mg, 0.262 mmol, 64%). ^1^H NMR (400 MHz DMSO-d^6^): δ 6.37 (m, 1H), 6.50 (m, 1H), 7.30 (m, 1H), 11.77 (br, 1H), 13.50 (br, 1H); ^13^C NMR (100 MHz DMSO-d^6^): δ 112.7, 115.0, 116.9, 121.9, 125.8, 127.2, 169.2, 194.8; HRMS: [M + H]^+^ calculated for C_8_H_7_N_2_OS_2_: 210.9994, found: 210.9997.


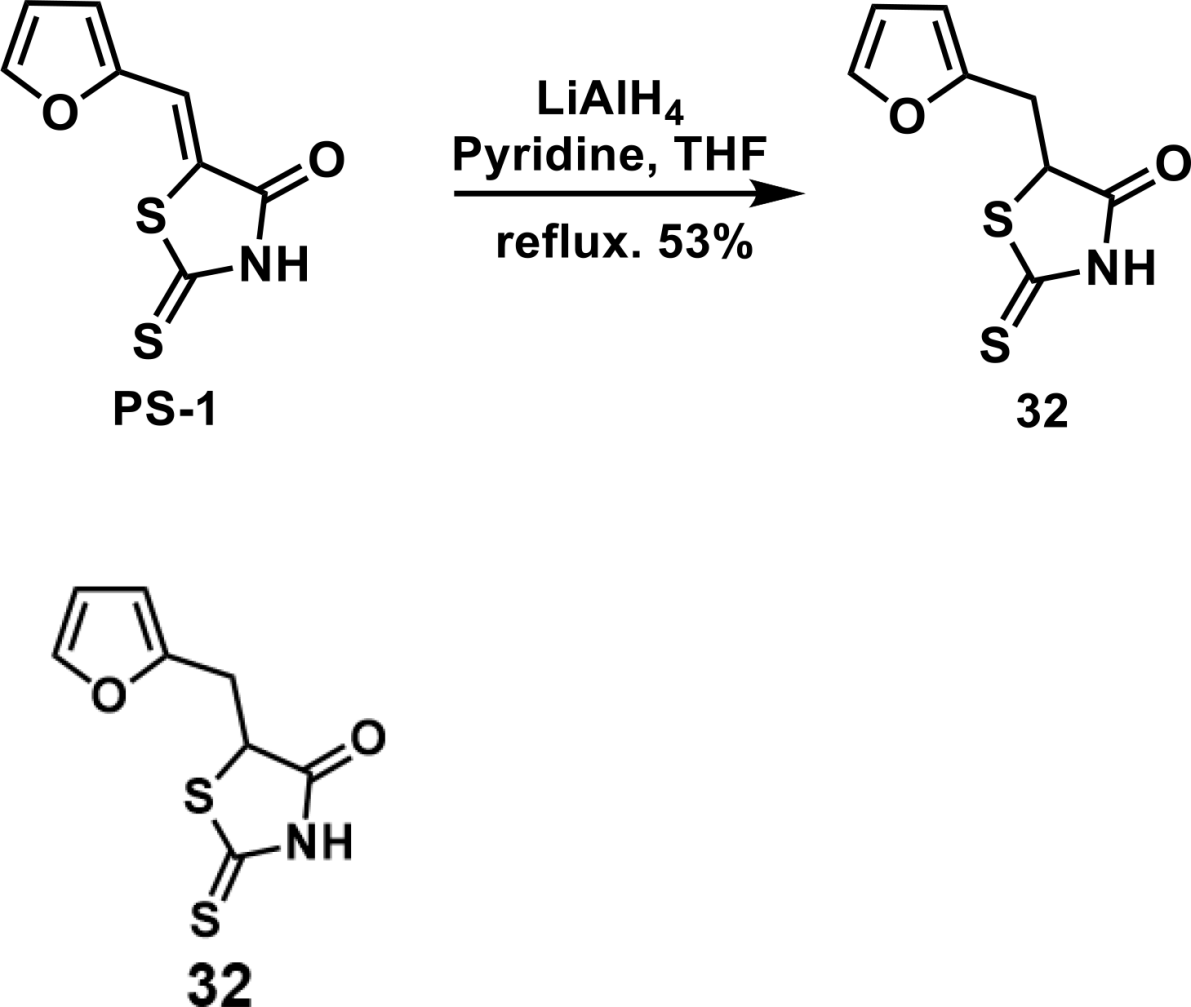
 5-(furan-2-ylmethyl)-2-thioxothiazolidin-4-one(32): To a solution of PS-1 (50 mg, 0.24 mmol) in THF/pyridine(1:1, 1 mL) was added 260 uL of LiAlH_4_ solution in THF(2 M). The mixture was stirred at reflux under the atmosphere of argon overnight. Then, the mixture was quenched by slowly adding 2M HCl solution. The mixture was extracted by ethyl acetate for 3 times. The combined organic layer was washed with brine, dried over *anhyd.* Na_2_SO_4_ and concentrated *in vacuo*. The residue was purified by column chromatography to give 32 as a brown solid (26.8 mg, 0.126 mmol, 53%). ^1^H NMR (400 MHz CDCl_3_): δ 3.28 (dd, *J* = 15.6 Hz, 9.6 Hz, 1H), 3.55 (dd, *J* = 15.6 Hz, 4 Hz, 1H), 4.66(dd, *J* = 9.6 Hz, 4 Hz, 1H), 6.18 (d, *J* = 3.6 Hz, 1H), 6.32 (dd, *J* = 3.2 Hz, 1.6 Hz, 1H), 7.36 (d, *J*=2 Hz 1H), 9.39 (br, 1H); ^13^C NMR (100 MHz CDCl_3_): δ 31.0, 53.7, 108.2, 110.6, 142.6, 149.4, 176.1, 200.0; HRMS: [M + H]^+^ calculated for C_8_H_8_NO_2_S_2_: 213.9991, found: 213.9993.

## Data availability statement

The original contributions presented in the study are included in the article/**Supplementary Material**. Further inquiries can be directed to the corresponding authors.

## Author contributions

YY, LiX, WX, LW, ML, LX, FH, SS, and LeX designed the research. YY, LiX, WX, LW, ML, LX, FH, and SS conducted the research. YY, LiX, and LeX analyzed the data. YY, LiX, and LeX wrote and edited the manuscript

## Funding

This work was supported by the National Natural Science Foundation of China (31872659, 32070301), the National Genetically Modified Organisms Breeding Major Projects (2016ZX08009002) and Exploration project of Center for Practical Courses in the Life Sciences of China Agricultural University (20210102, 20210103).

## Conflict of interest

The authors declare that the research was conducted in the absence of any commercial or financial relationships that could be construed as a potential conflict of interest.

## Publisher’s note

All claims expressed in this article are solely those of the authors and do not necessarily represent those of their affiliated organizations, or those of the publisher, the editors and the reviewers. Any product that may be evaluated in this article, or claim that may be made by its manufacturer, is not guaranteed or endorsed by the publisher.
