# Supplementary material for: Screening of protonstatin-1 (PS-1) analogs for improved inhibitors of plant plasma membrane H+-ATPase activity
Source: Front Plant Sci. 2022 Oct 12;13:973471. doi: 10.3389/fpls.2022.973471 (PMC9597486; doi:10.3389/fpls.2022.973471)

**Supporting information**

**Synthesis and Screening of Plasma Membrane H^+^-ATPase Inhibitors with Higher Affinity than PS-1**

Yongqing Yang^a, #, *^, Xiaohui Liu^b^^, #^, Xin Wang^b, #^, Wanjia Lv^a, #^, Xiao Liu ^a, #^, Liang Ma, Haiqi Fu, Shu Song, and Xiaoguang Lei^b, *^

**Sup Table 1.**

| + PS-2 (μM) | Km (mM) | Vmax (μM Pi mg^-1^ protein min^-1^) |
| --- | --- | --- |
| + 0 | 0.64 +/- 0.03 | 4.59 +/- 0.09 |
| 0.25 | 0.68 +/- 0.06 | 3.52 +/- 0.21 |
| 0.5 | 0.68 +/- 0.09 | 2.84 +/- 0.27 |
| 1 | 0.61 +/- 0.21 | 1.69 +/- 0.08 |
| 2.5 | 0.56 +/- 0.26 | 0.58 +/- 0.13 |

**Sup Table 1. Effect of PS-2 on the ATP hydrolysis of the PM H^+^-ATPase.**

In the Sup Table 1, the PM H^+^-ATPase hydrolytic activity was measured with various concentrations of ATP (0, 0.25, 0.5, 1, 2, 3, 4 mM) in the presence of various concentrations of PS-2 (0, 0.25, 0.5, 1, 2.5 μM) and the kinetic constants (Km values, Vmax) were determined from nonlinear regression of the Michaelis-Menten equation. Values are mean ± SD (n = 5).

**Sup Table 2.**

| + PS-1 (μM) | Km (mM) | Vmax (μM Pi mg^-1^ protein min^-1^) |
| --- | --- | --- |
| 0 | 0.66 +/- 0.03 | 4.50 +/- 0.11 |
| 1 | 0.62 +/- 0.03 | 3.62 +/- 0.08 |
| 2.5 | 0.65 +/- 0.08 | 2.94 +/- 0.17 |
| 5 | 0.58+/- 0.12 | 2.10 +/- 0.14 |
| 10 | 0.52 +/- 0.34 | 1.09 +/- 0.24 |

**Sup Table 2. Effect of PS-1 on the ATP hydrolysis of the PM H^+^-ATPase.**

In the Sup Table 2, the PM H^+^-ATPase hydrolytic activity was measured with various concentrations of ATP (0, 0.25, 0.5, 1, 2, 3, 4 mM) in the presence of various concentrations of PS-1 (0, 1, 2.5, 5, 10 μM) and the kinetic constants (Km values, Vmax) were determined from nonlinear regression of the Michaelis-Menten equation. Values are mean ± SD (n = 5).

**Sup Table 3.**

| + 307-2 (μM) | Km (mM) | Vmax (μM Pi mg^-1^ protein min^-1^) |
| --- | --- | --- |
| 0 | 0.65 +/- 0.05 | 4.49 +/- 0.13 |
| 10 | 0.62 +/- 0.07 | 4.38 +/- 0.29 |

**Sup Table 3. Effect of 307-2 on the ATP hydrolysis of the PM H^+^-ATPase.**

In the Sup Table 3, the PM H^+^-ATPase hydrolytic activity was measured with various concentrations of ATP (0, 0.25, 0.5, 1, 2, 3, 4 mM) in the presence PS-1 of 10 μM and the kinetic constants (Km values, Vmax) were determined from nonlinear regression of the Michaelis-Menten equation. Values are mean ± SD (n = 5).

**Sup Figure 1.**

**
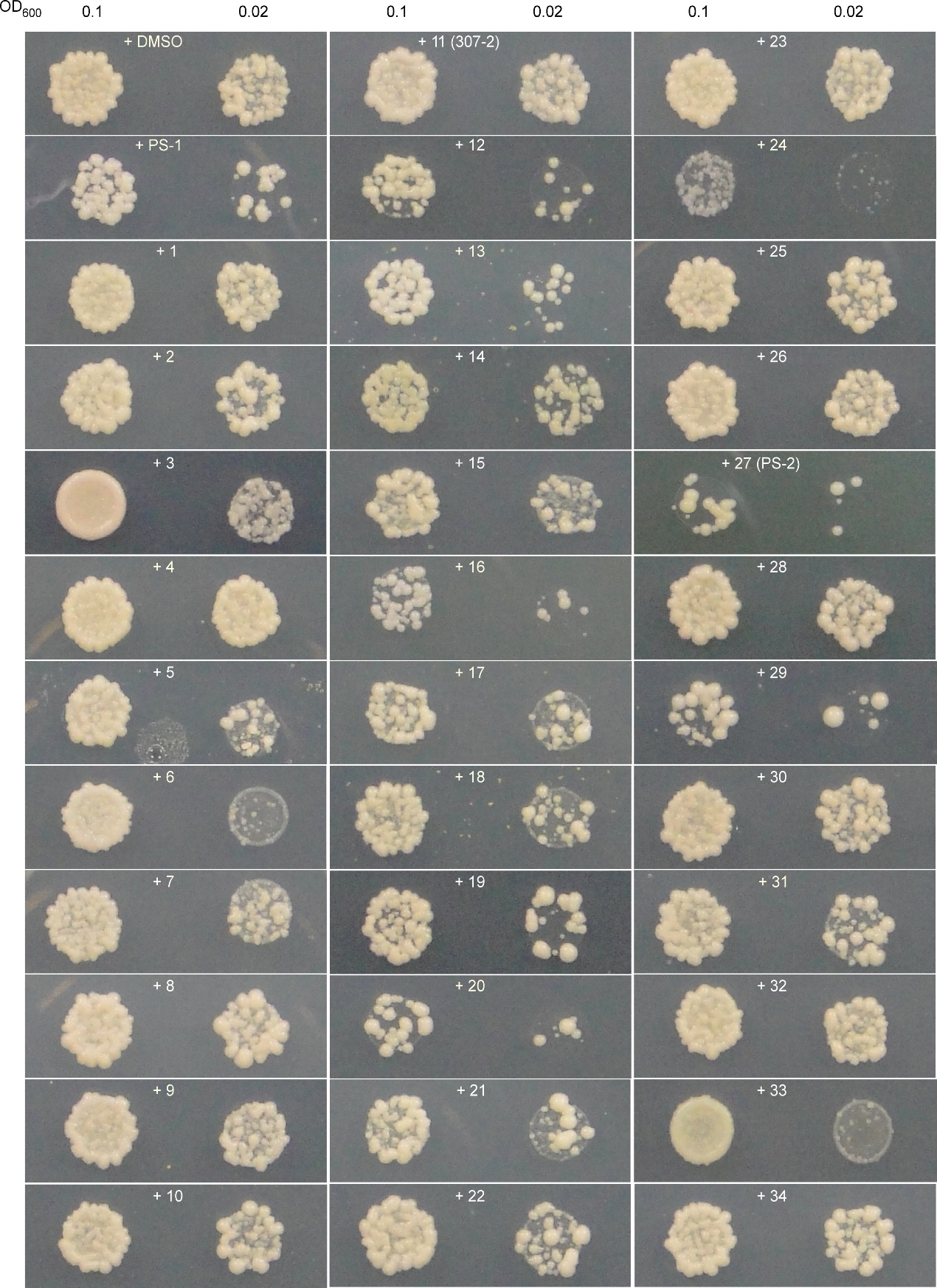
** **Sup Figure 1. Screening PS-1 analogs for the ability to inhibit PM H^+^-ATPase activity in yeast strain RS72 cells expressing Arabidopsis AHA2**

Effects of PS-1 analogs on the growth of yeast strain RS72-AHA2 on glucose medium at pH 6.5. Yeast strain RS-72 is dependent on the activity of AHA2, which is expressed in cells grown on glucose medium. The yeast cells were diluted in glucose medium, and 5 μL aliquots of two concentrations of cells were spotted onto solid glucose medium (OD_600_ = 0.1 and 0.02) in the presence of the DMSO control (0.1% [v/v]) or 10 μM PS-1 or PS-1 analog. The yeast cells were incubated at 28°C for 3 d prior to photography.

**Sup Figure 2.**

**
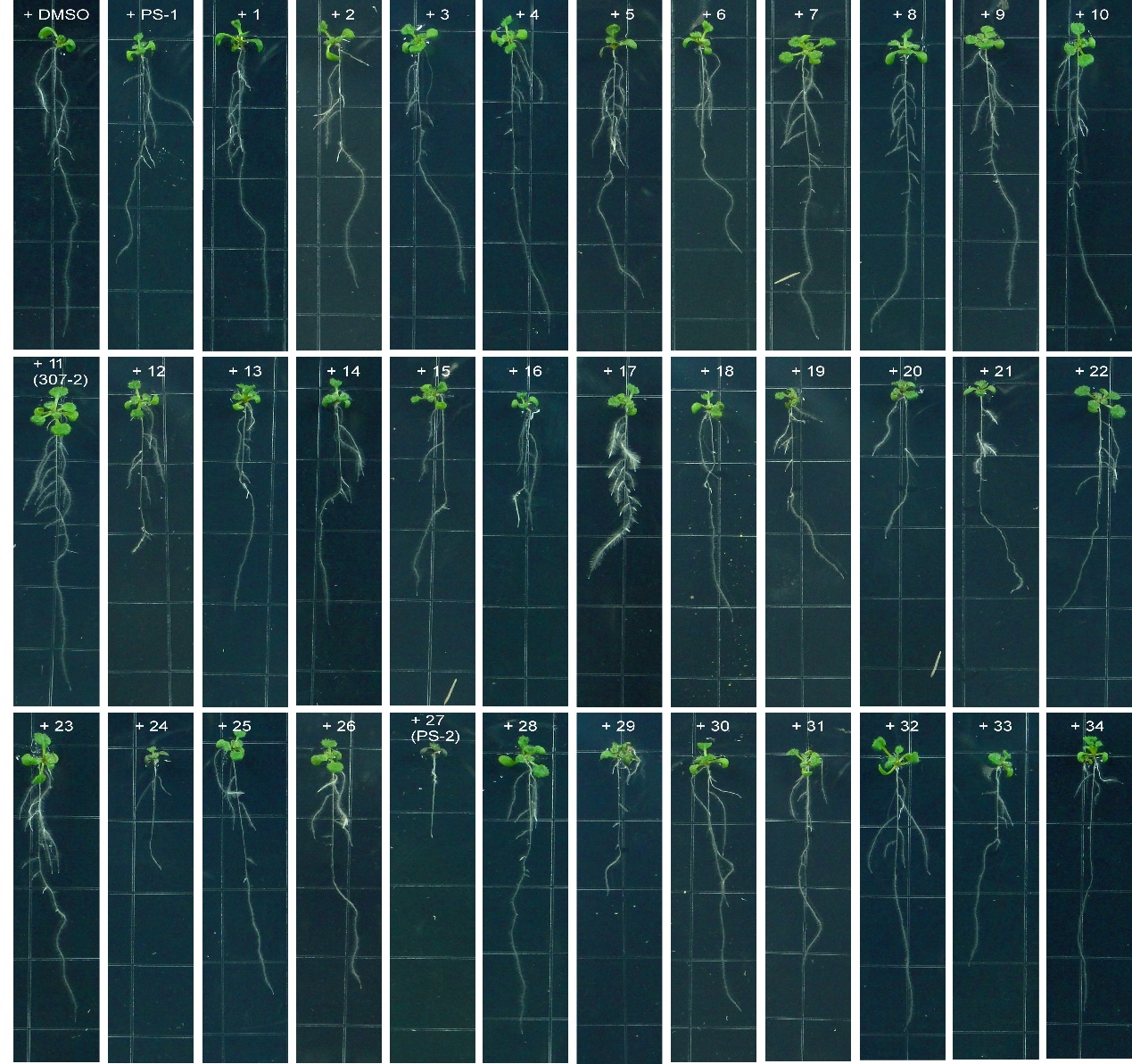
Sup Figure 2. Effects of** **PS-1 analogs on Arabidopsis seedling growth**

Representative photographs of PS-1 analog-treated seedlings. Five-day-old seedlings grown on MS medium, pH 5.8, were transferred to plates containing 2.5 mM PS-1 or PS-1 analog (or 0.1% [v/v] DMSO as a control) and incubated for 7 d at 22°C for 7 d prior to photography.

**NMR spectra**


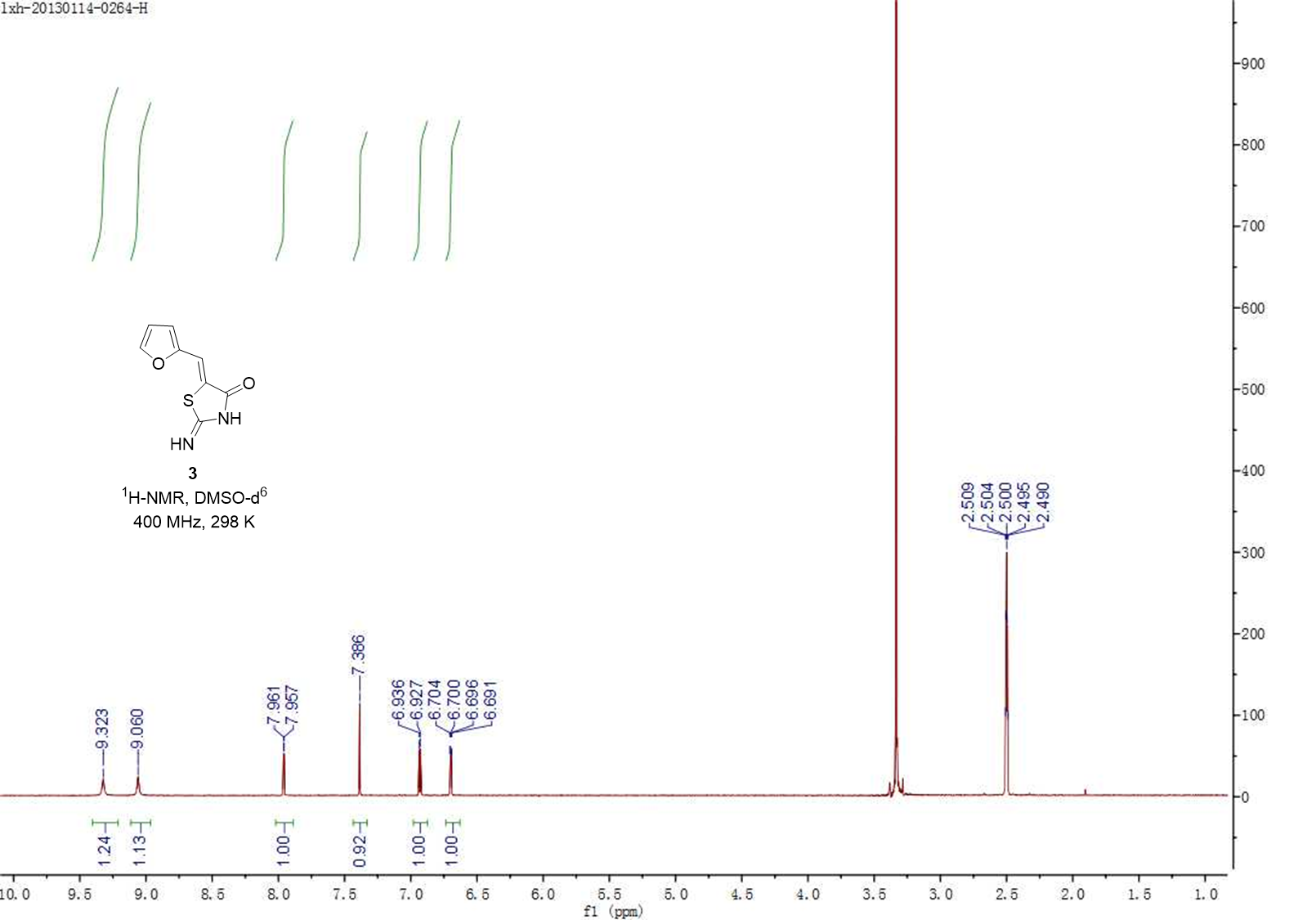


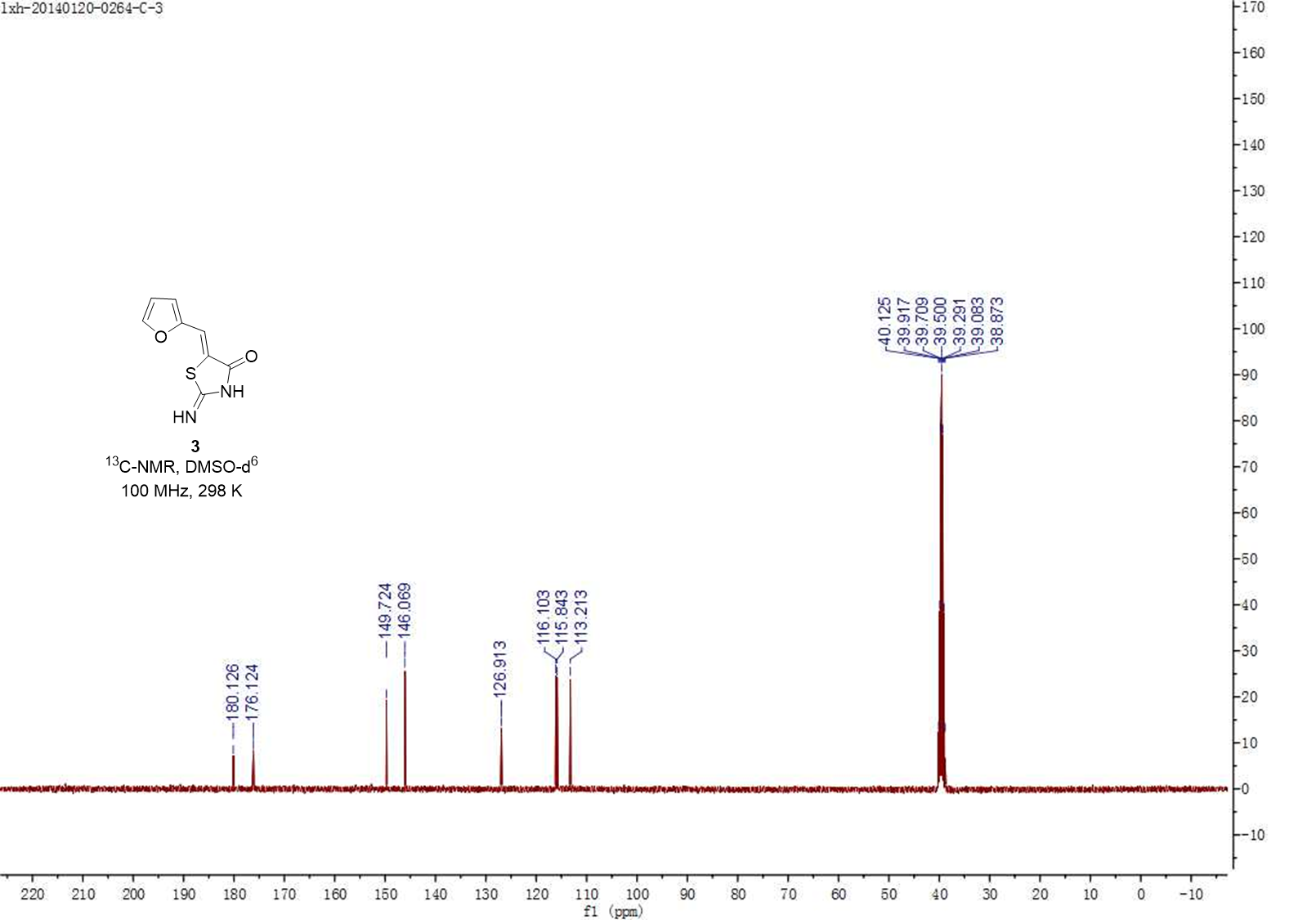


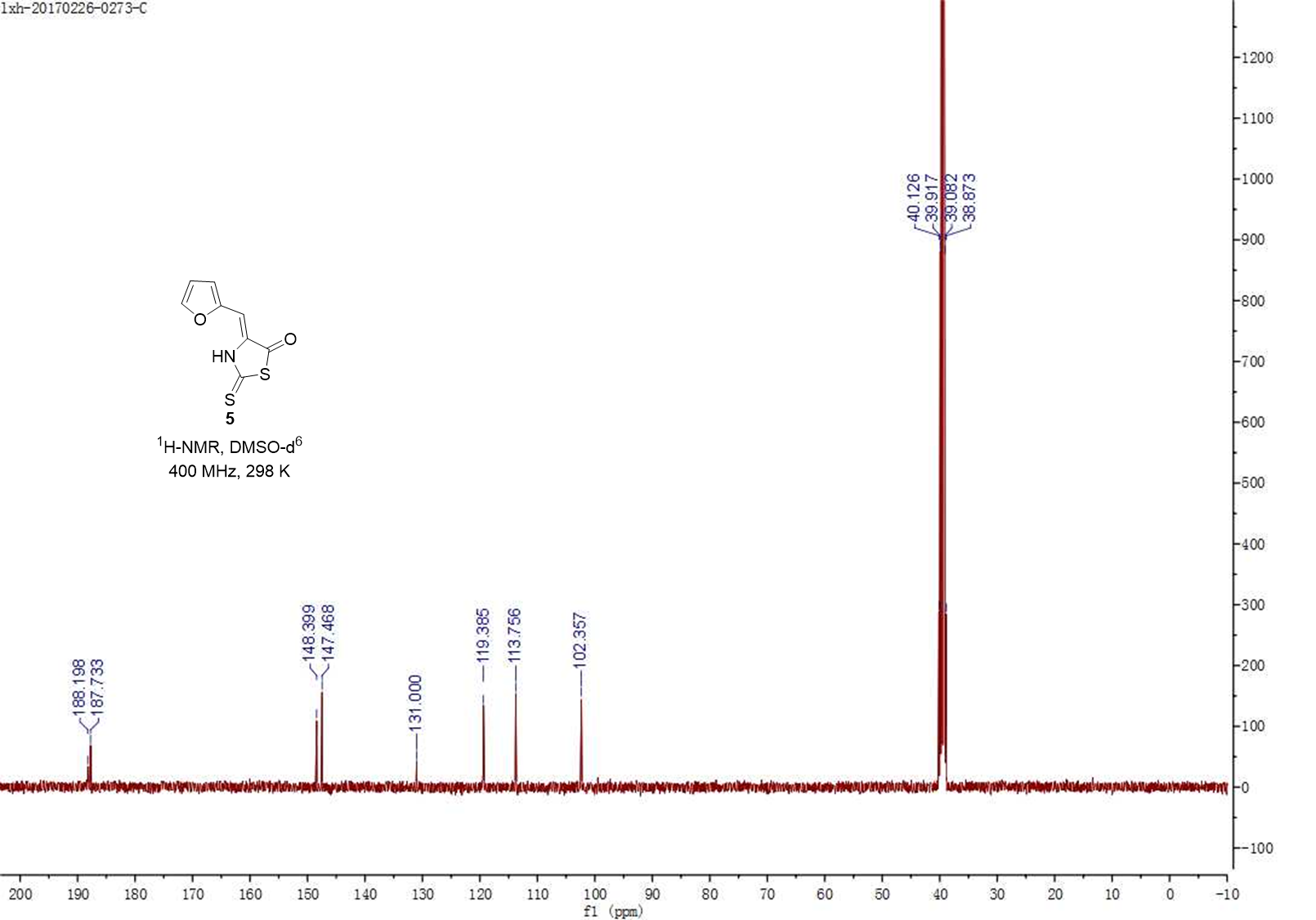

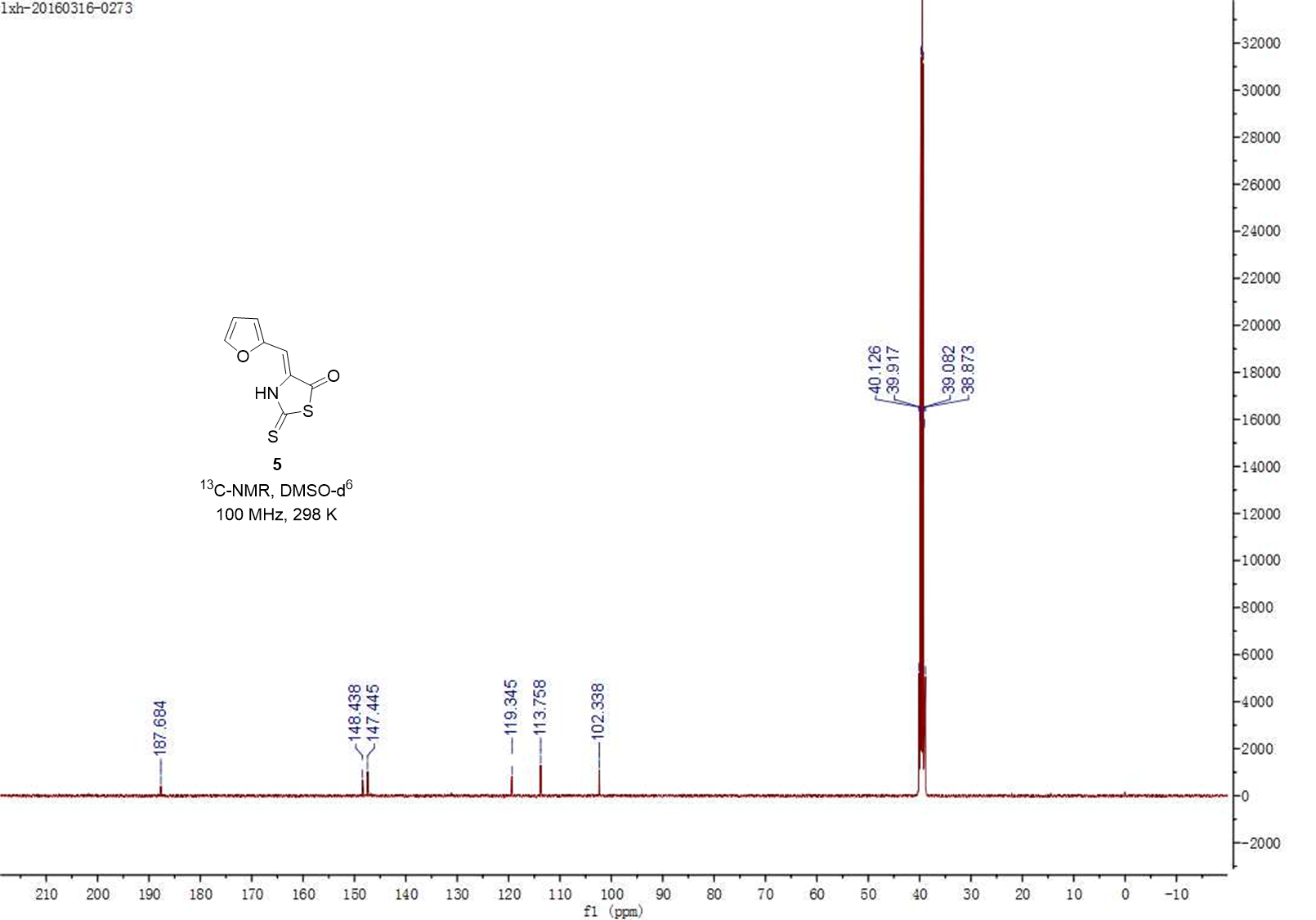

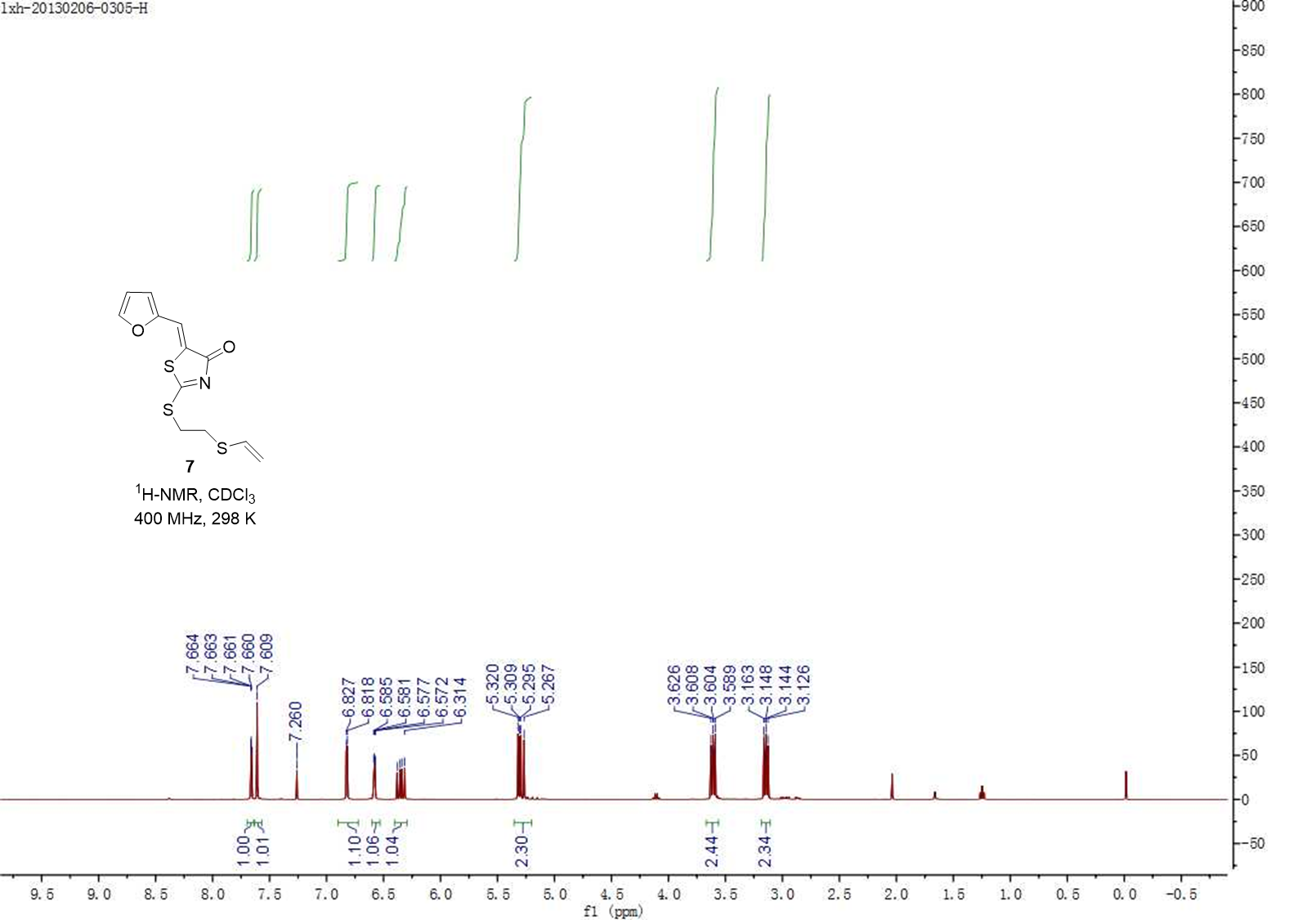

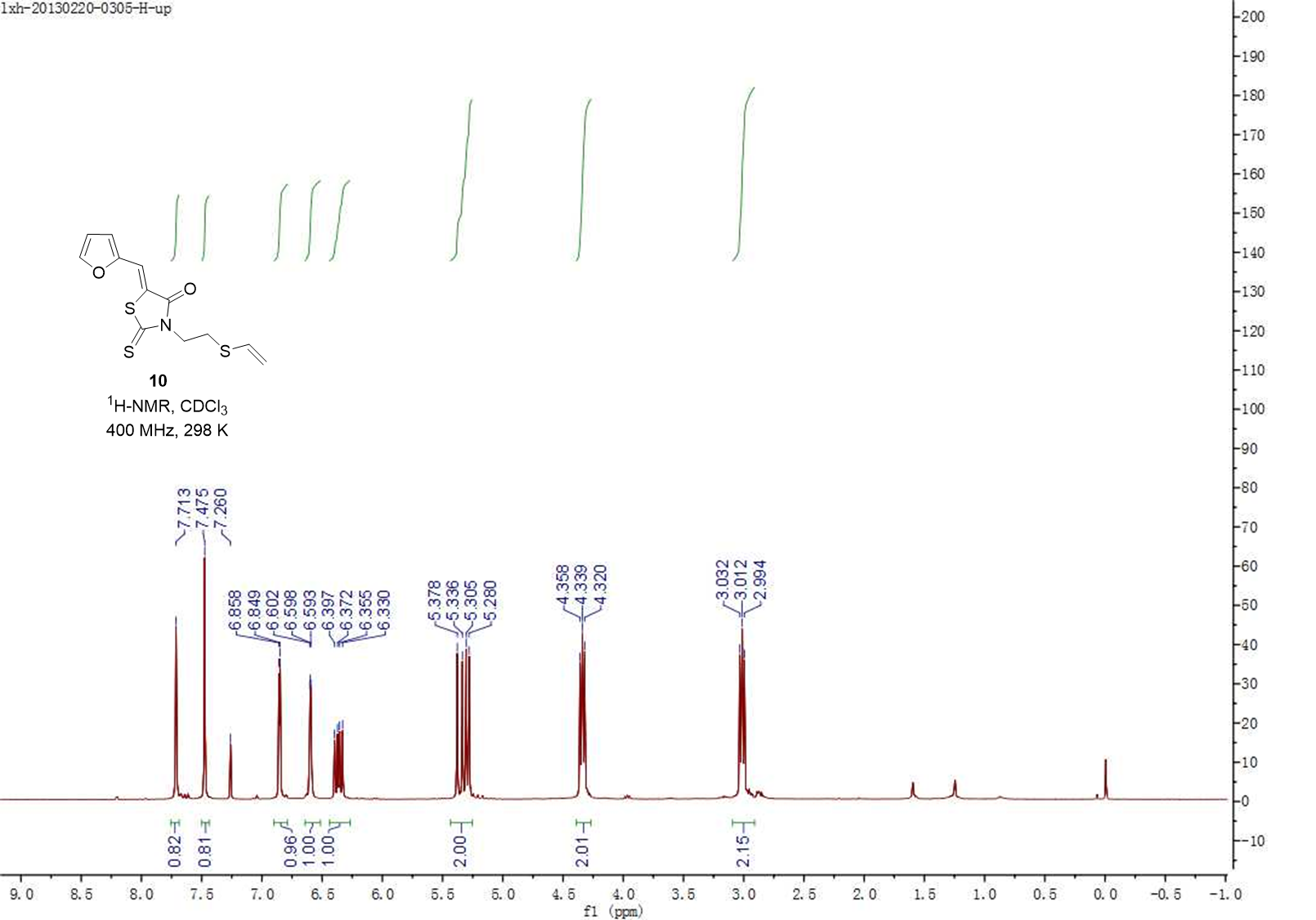

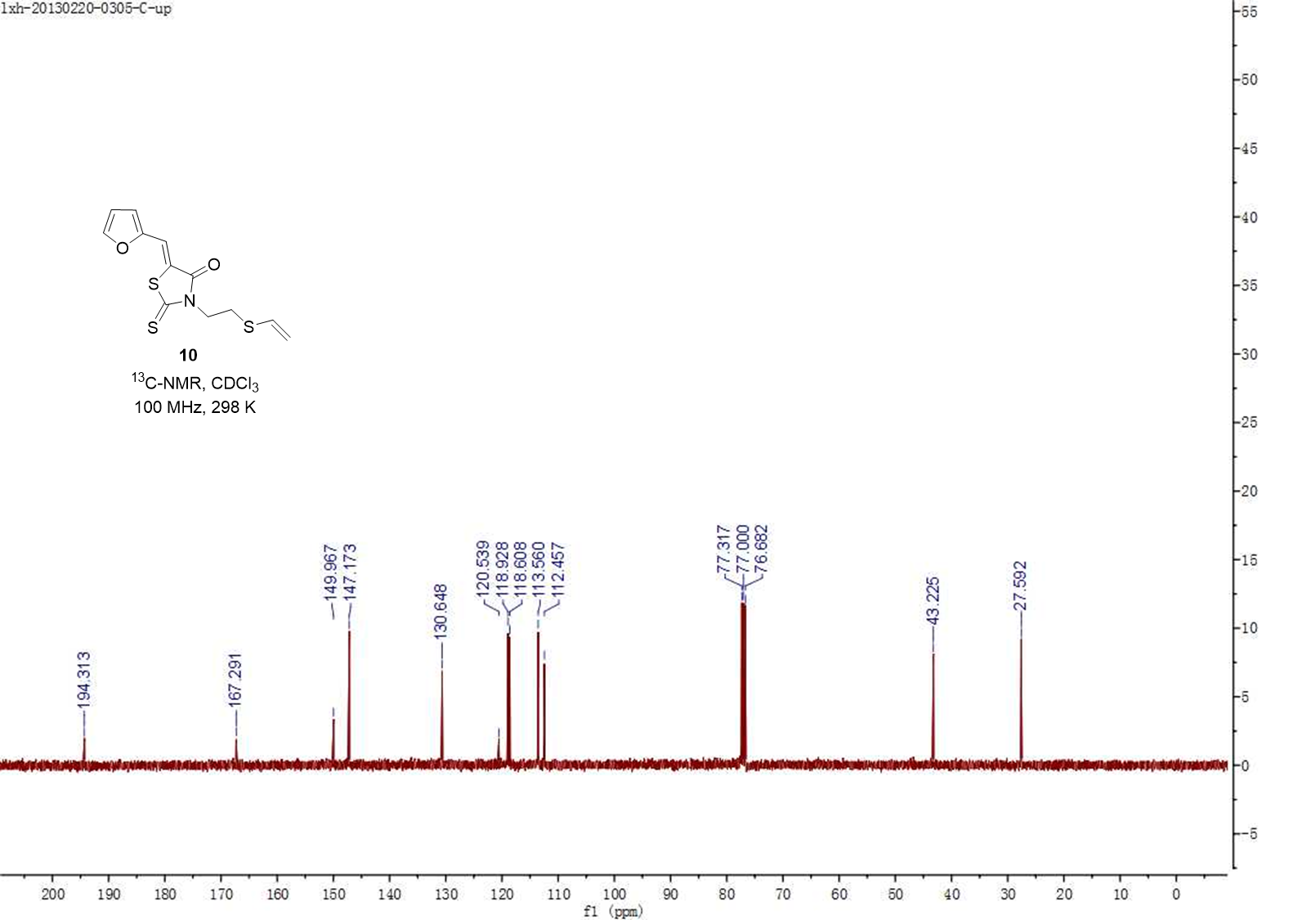

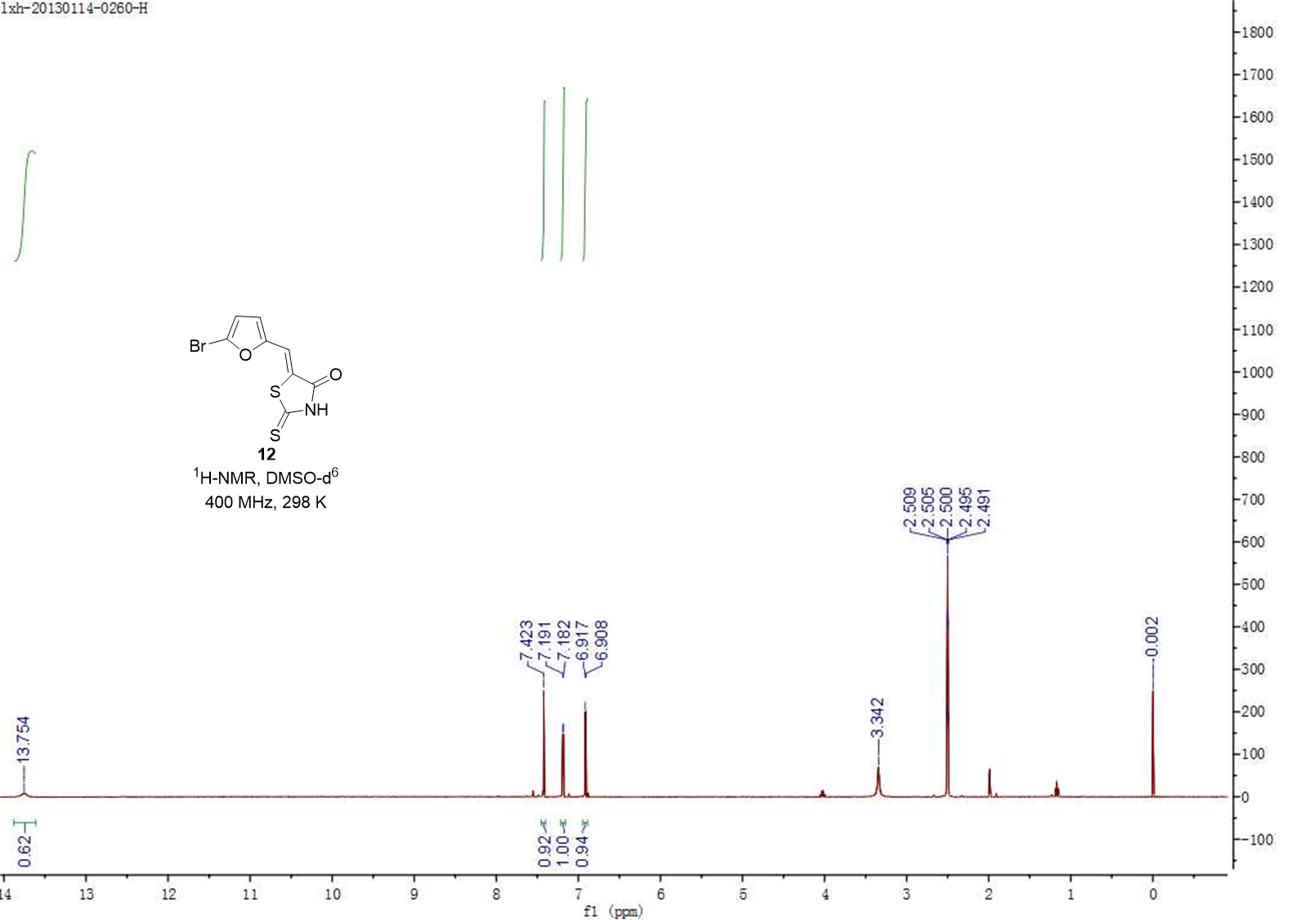

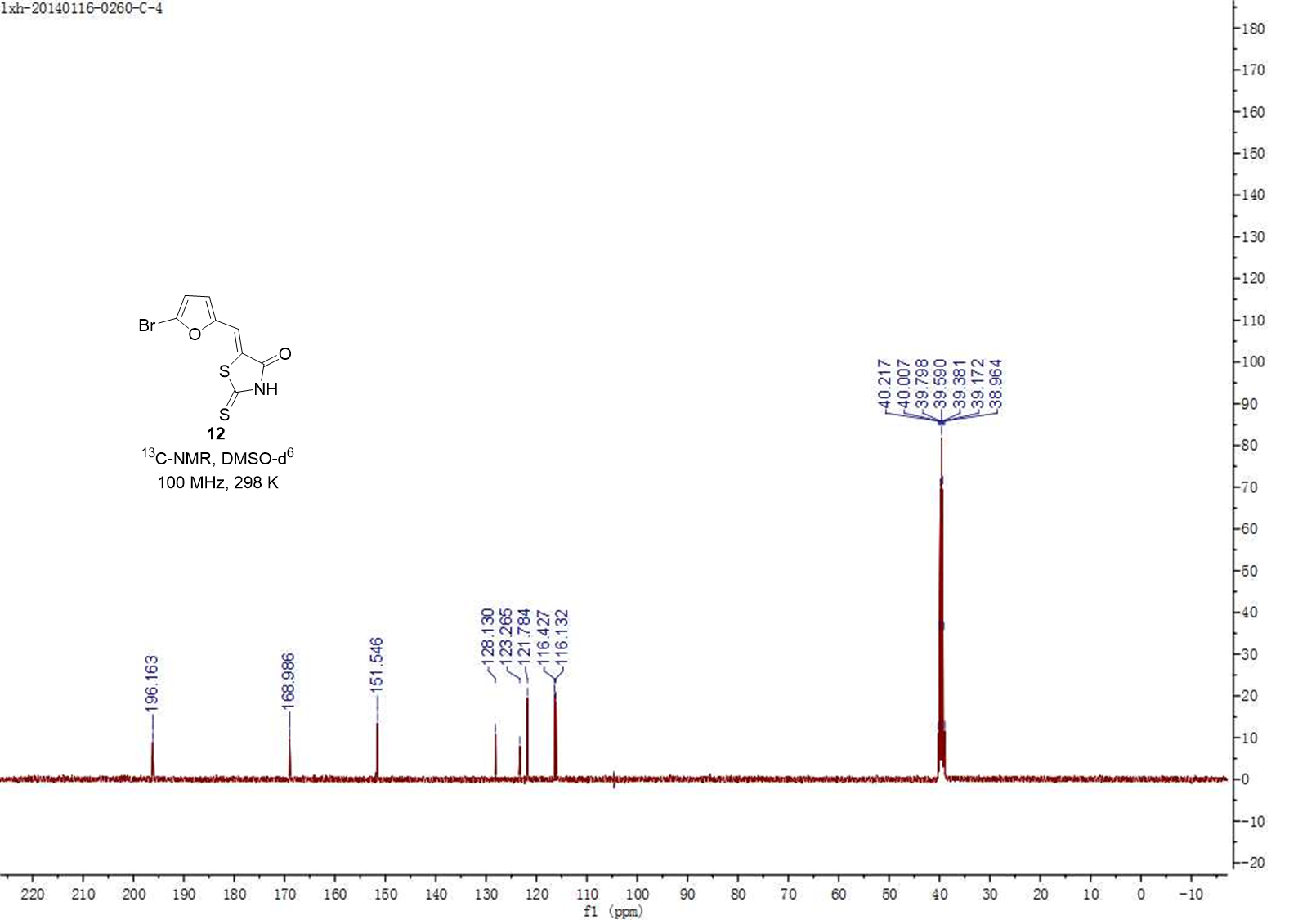

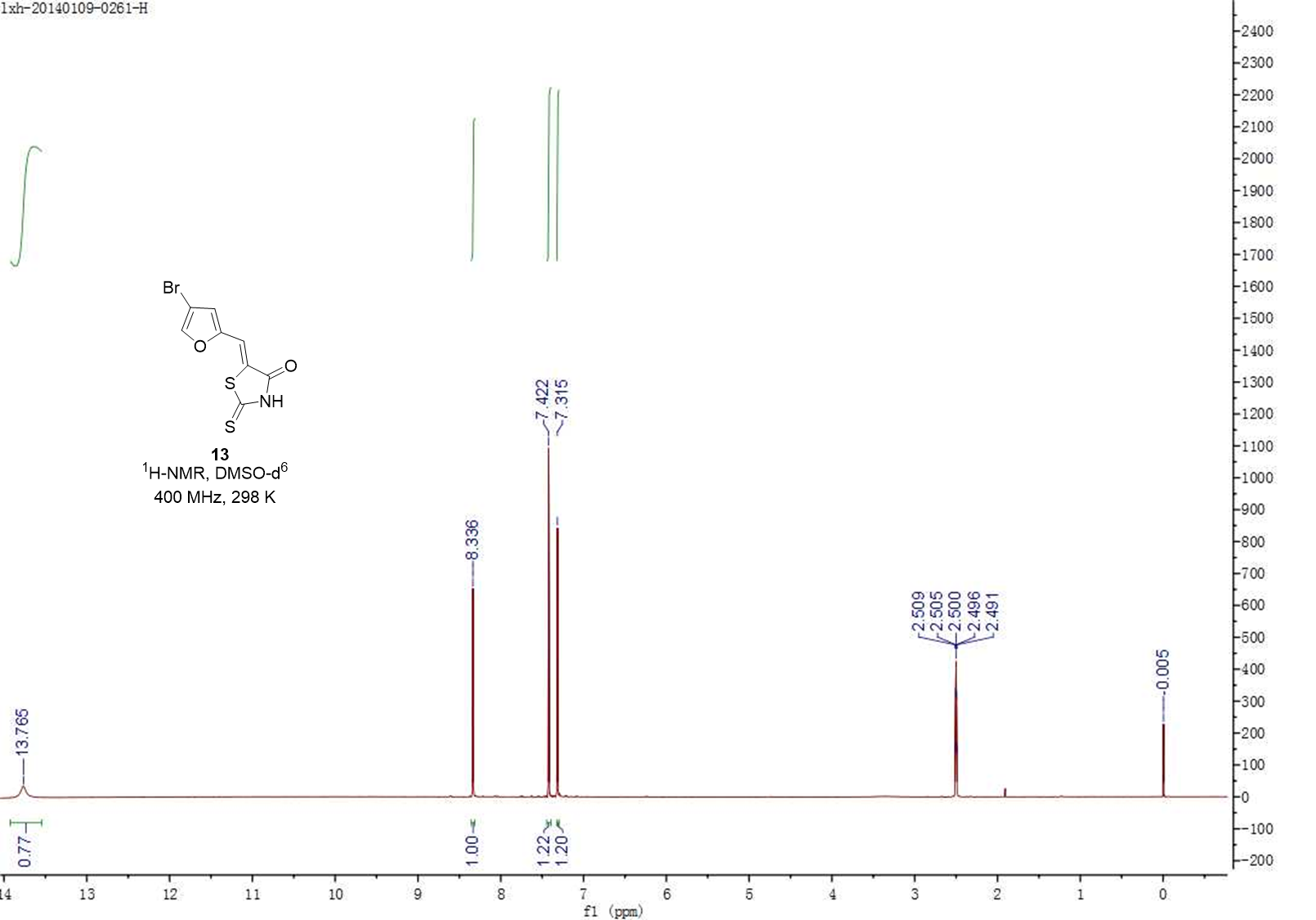

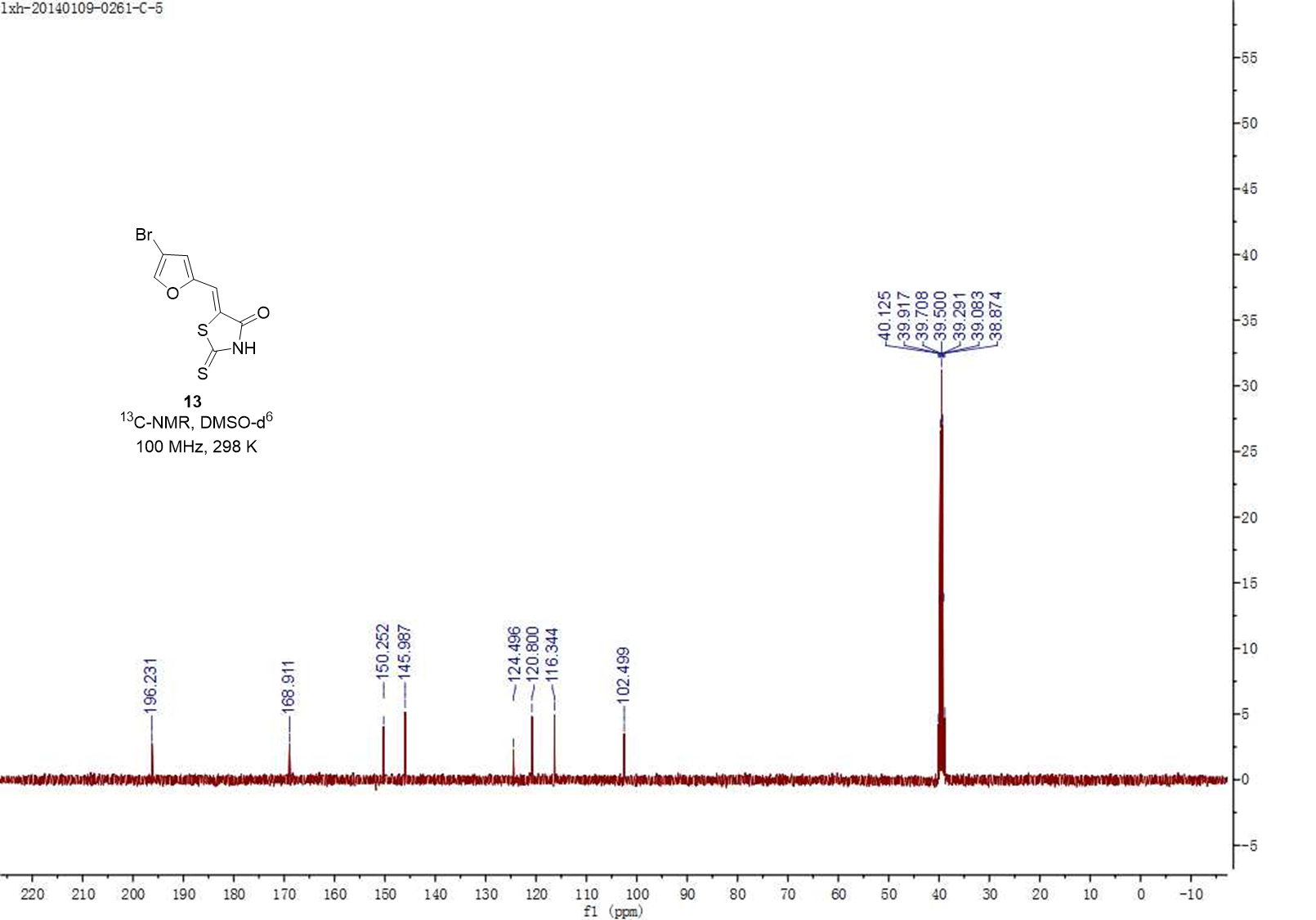

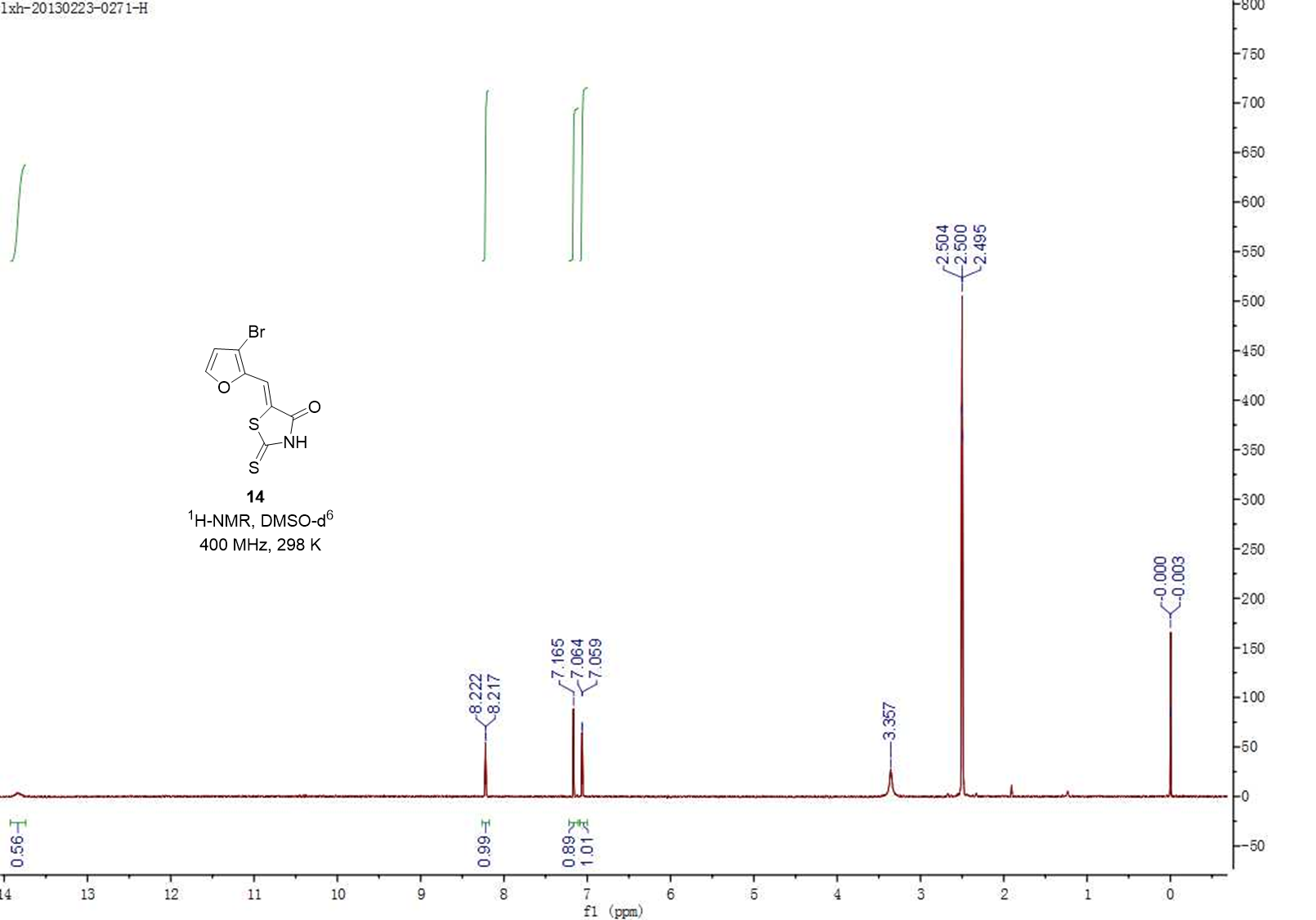

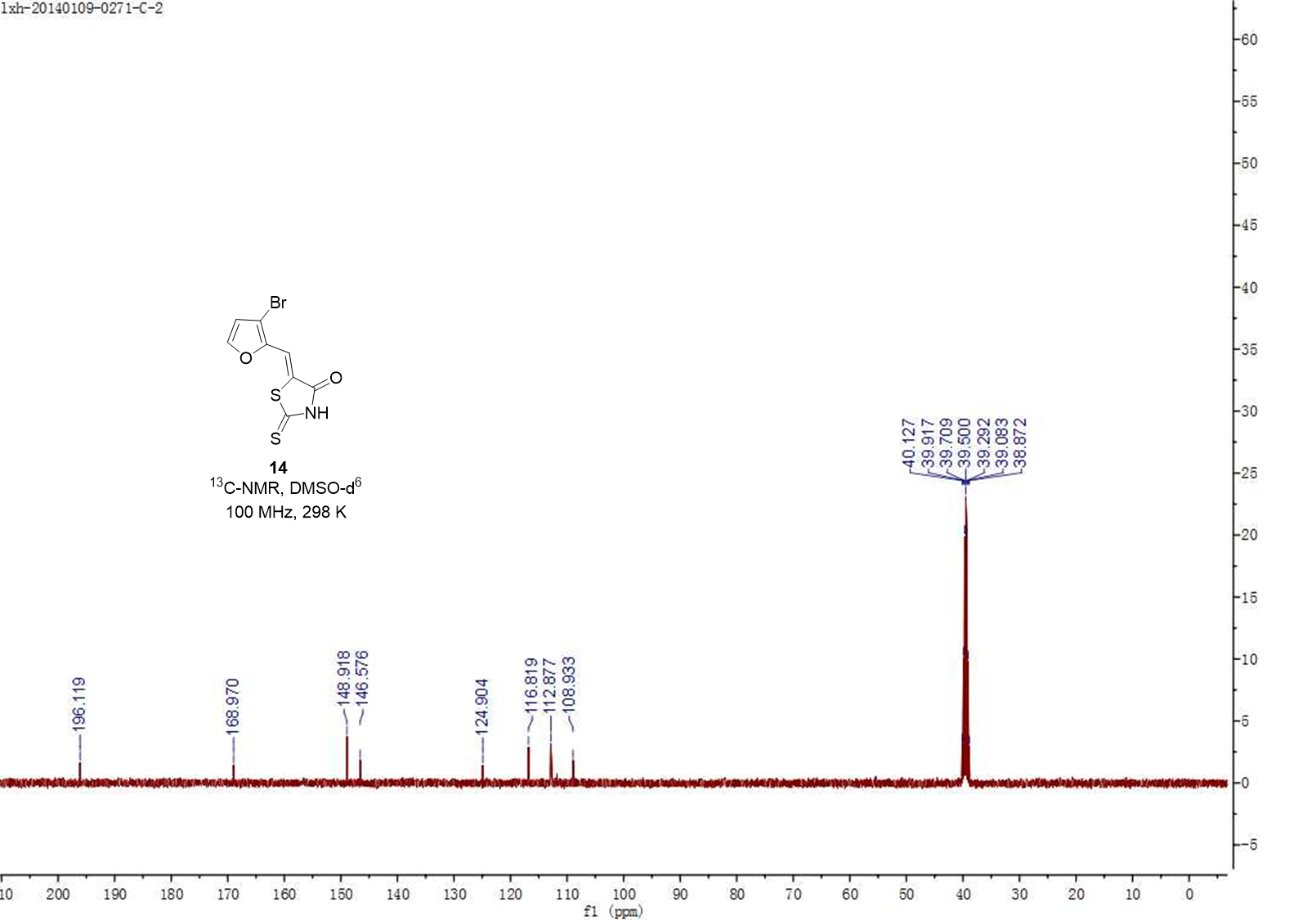

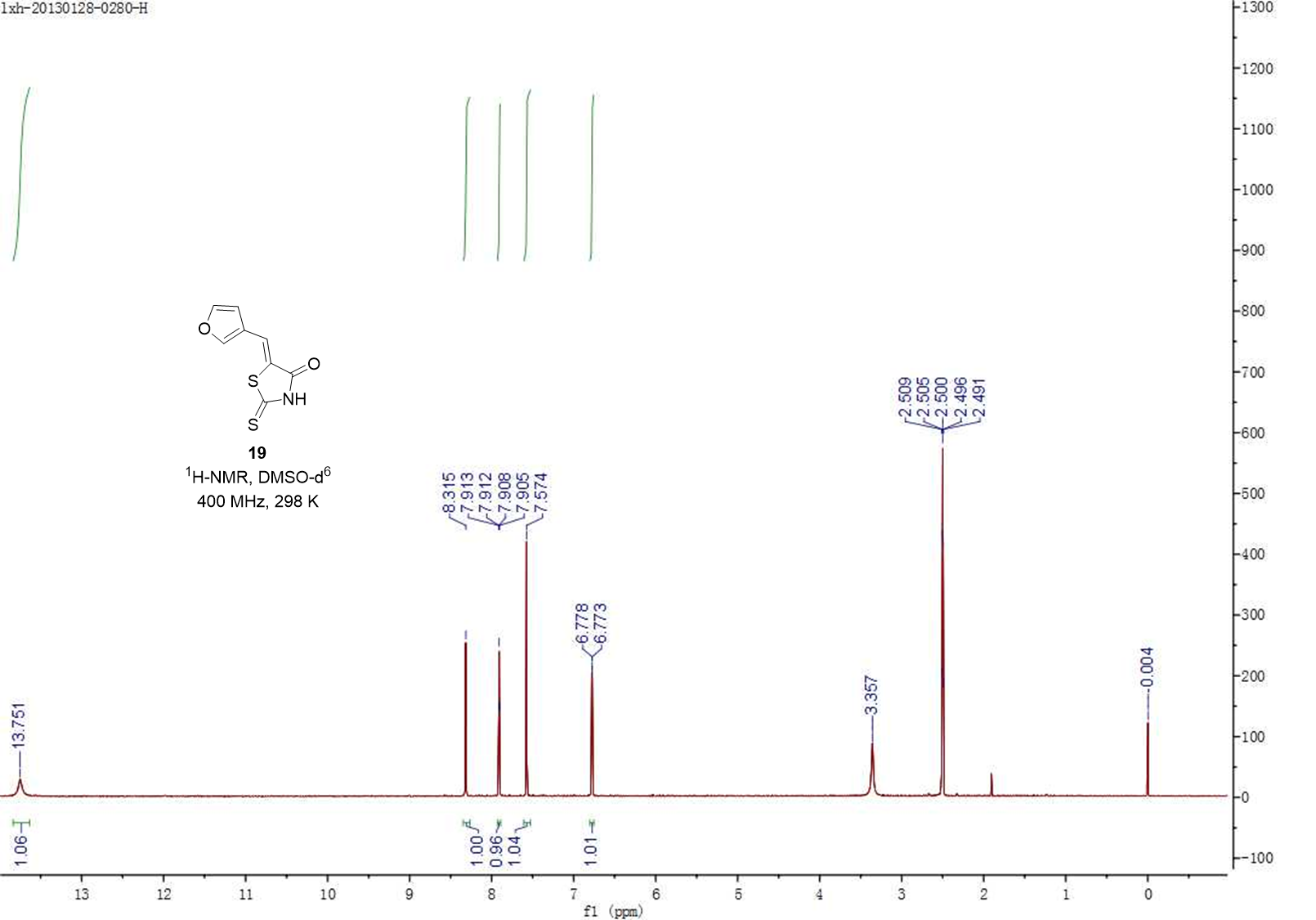

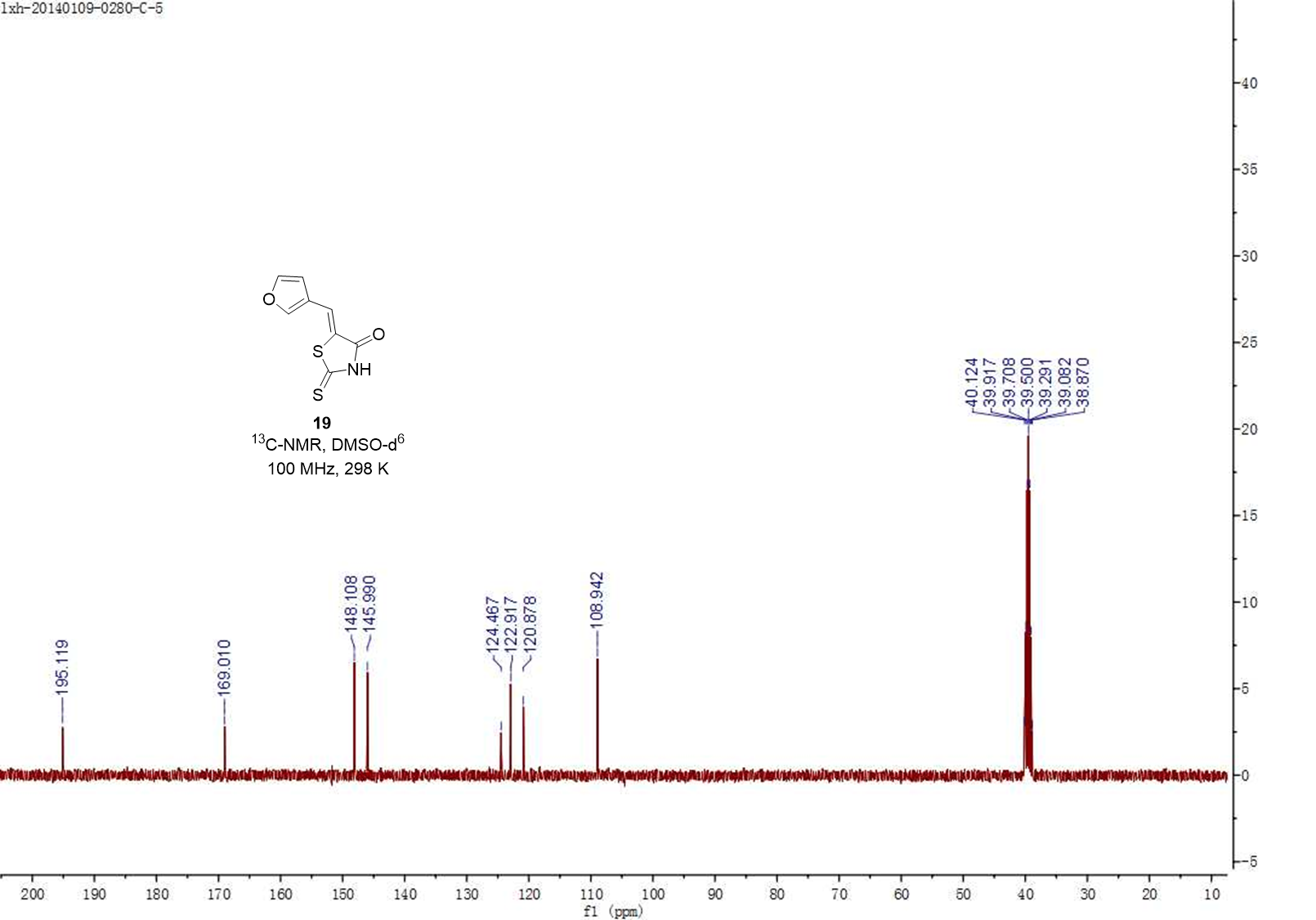

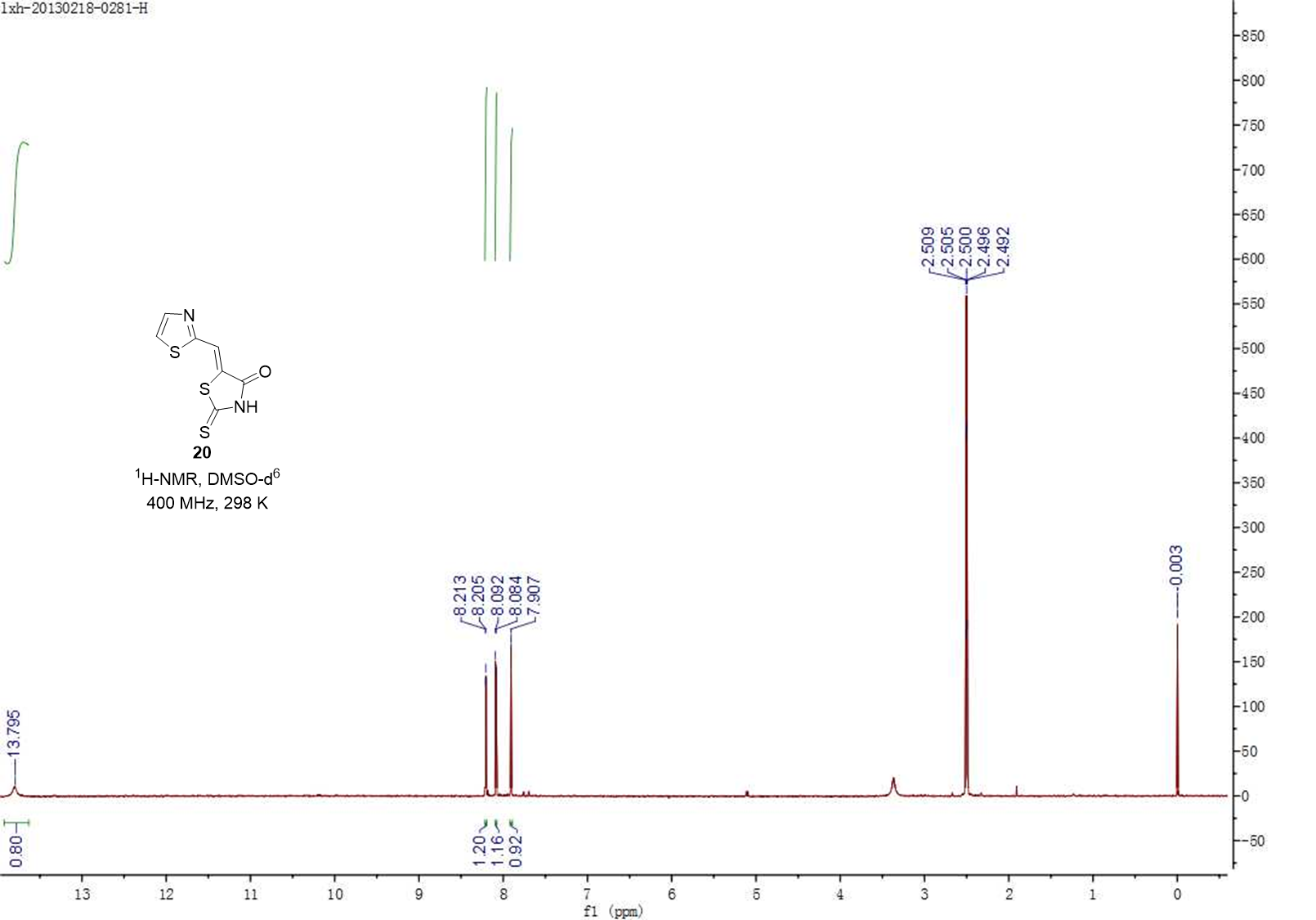

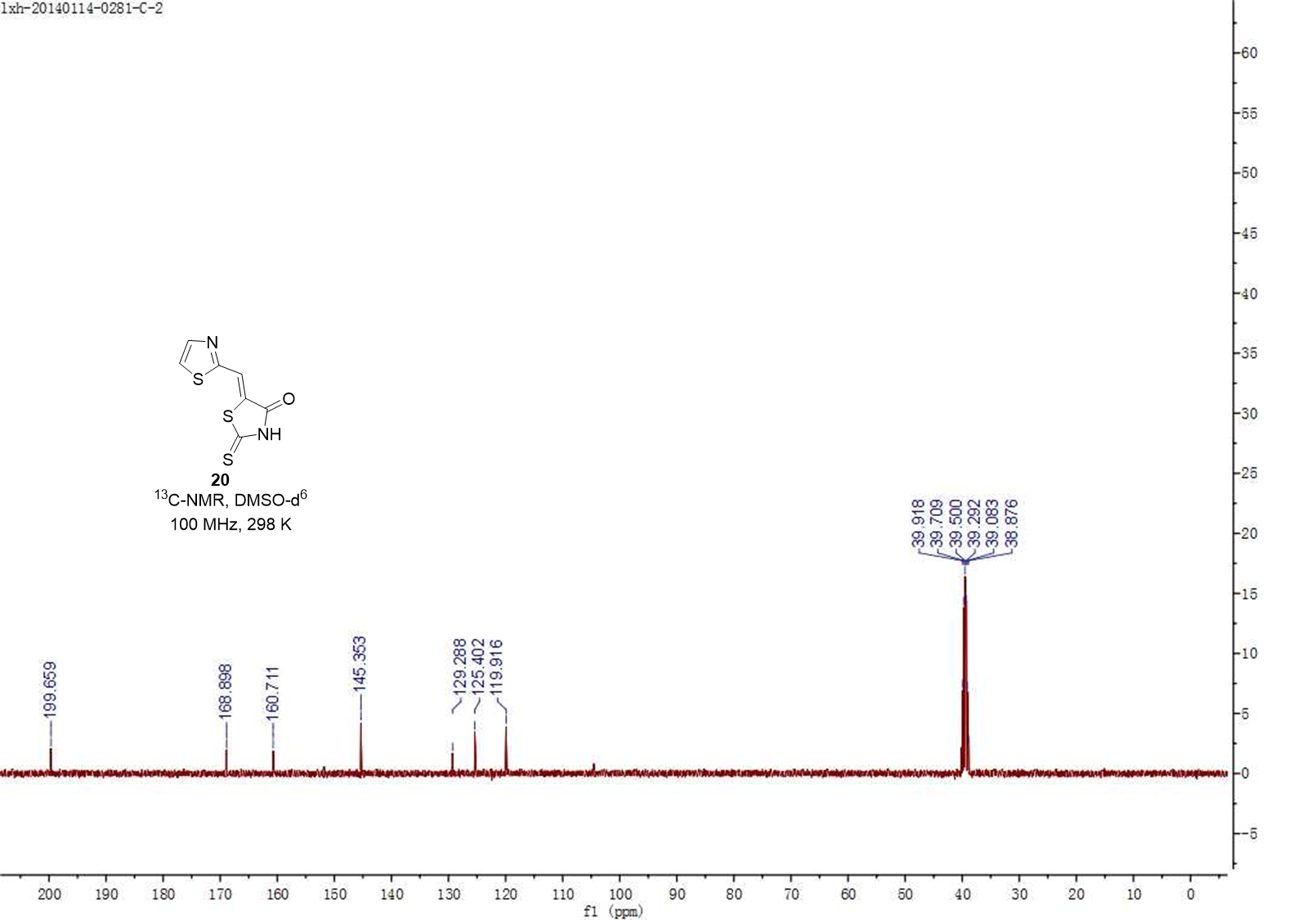

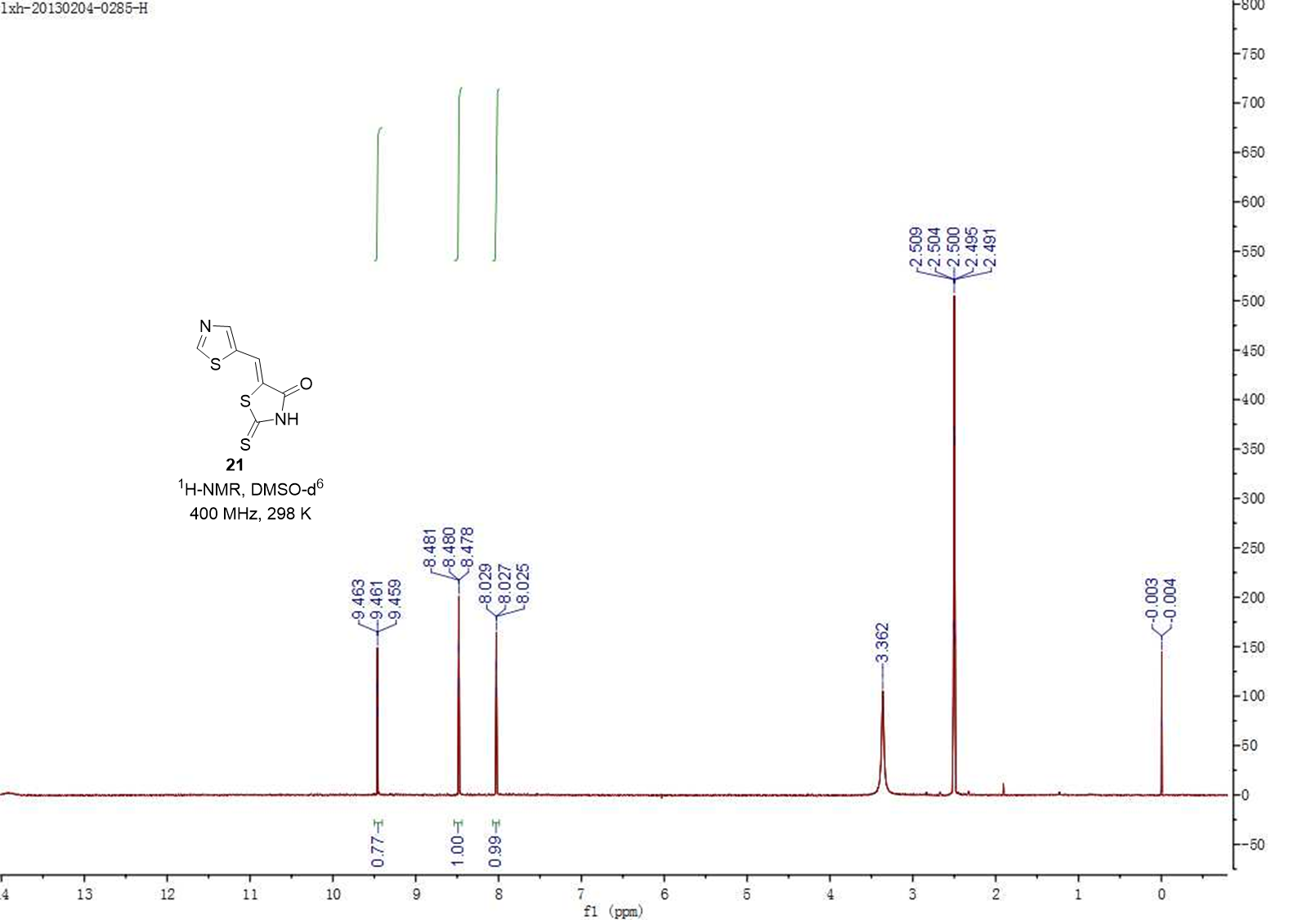

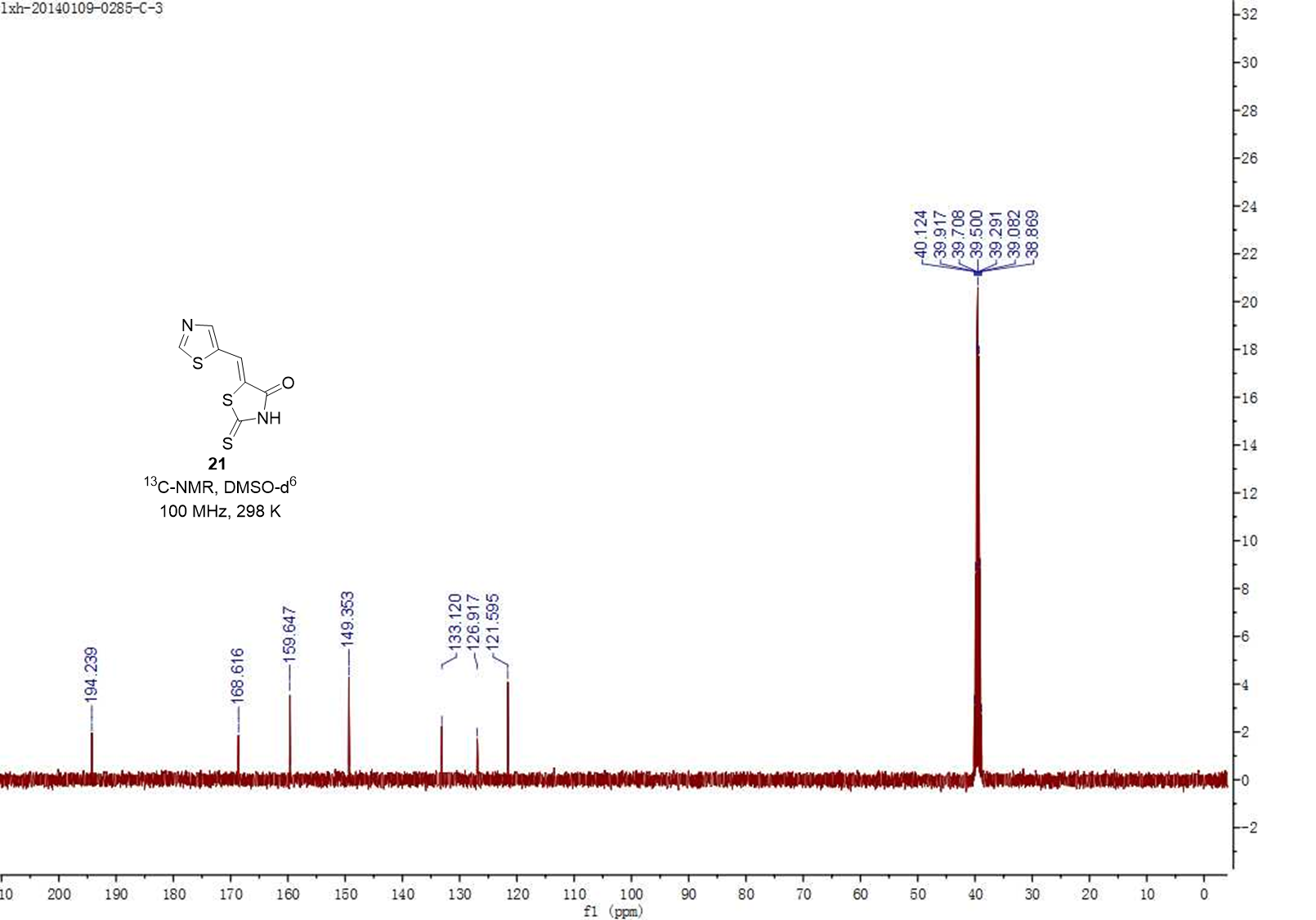

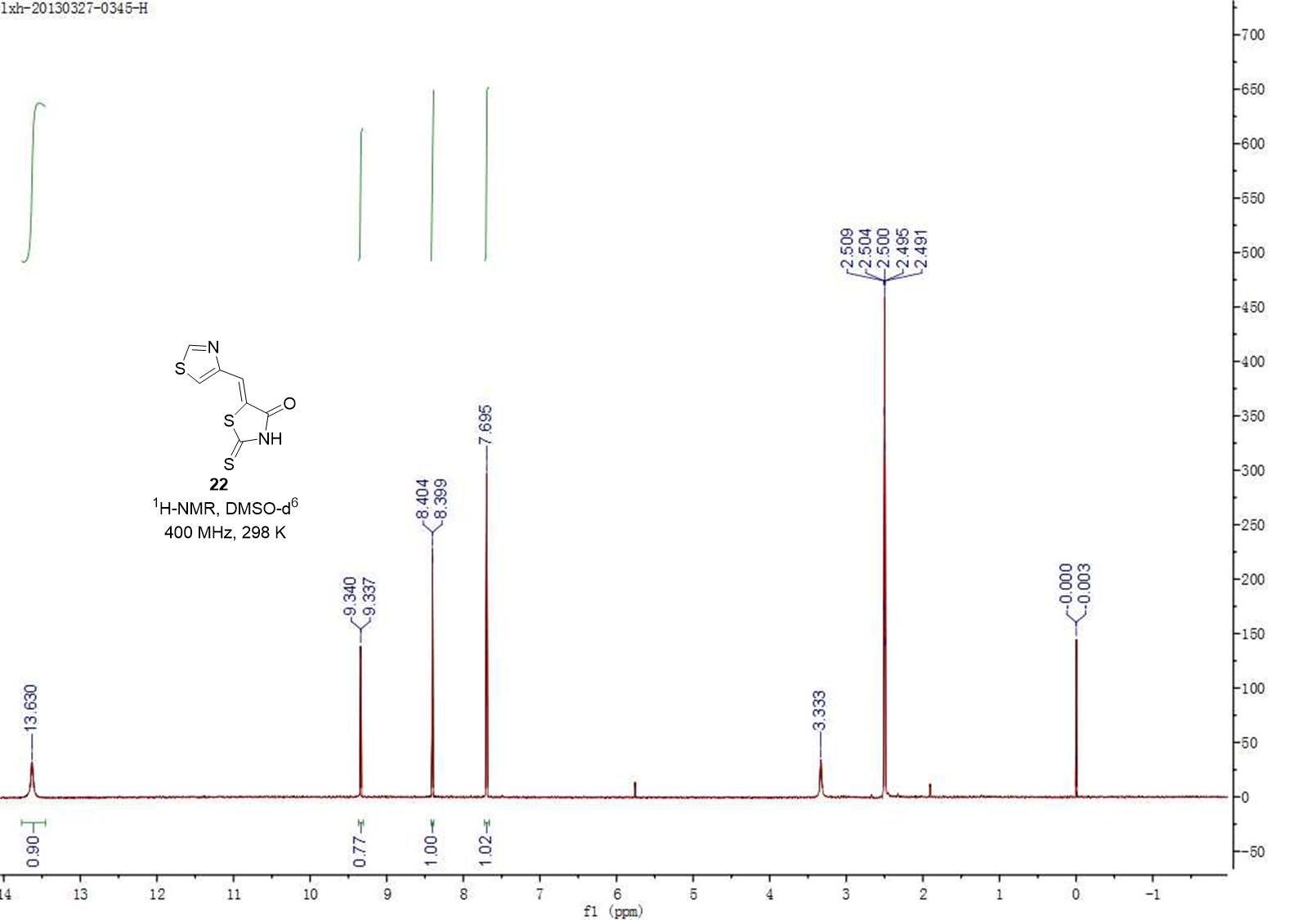

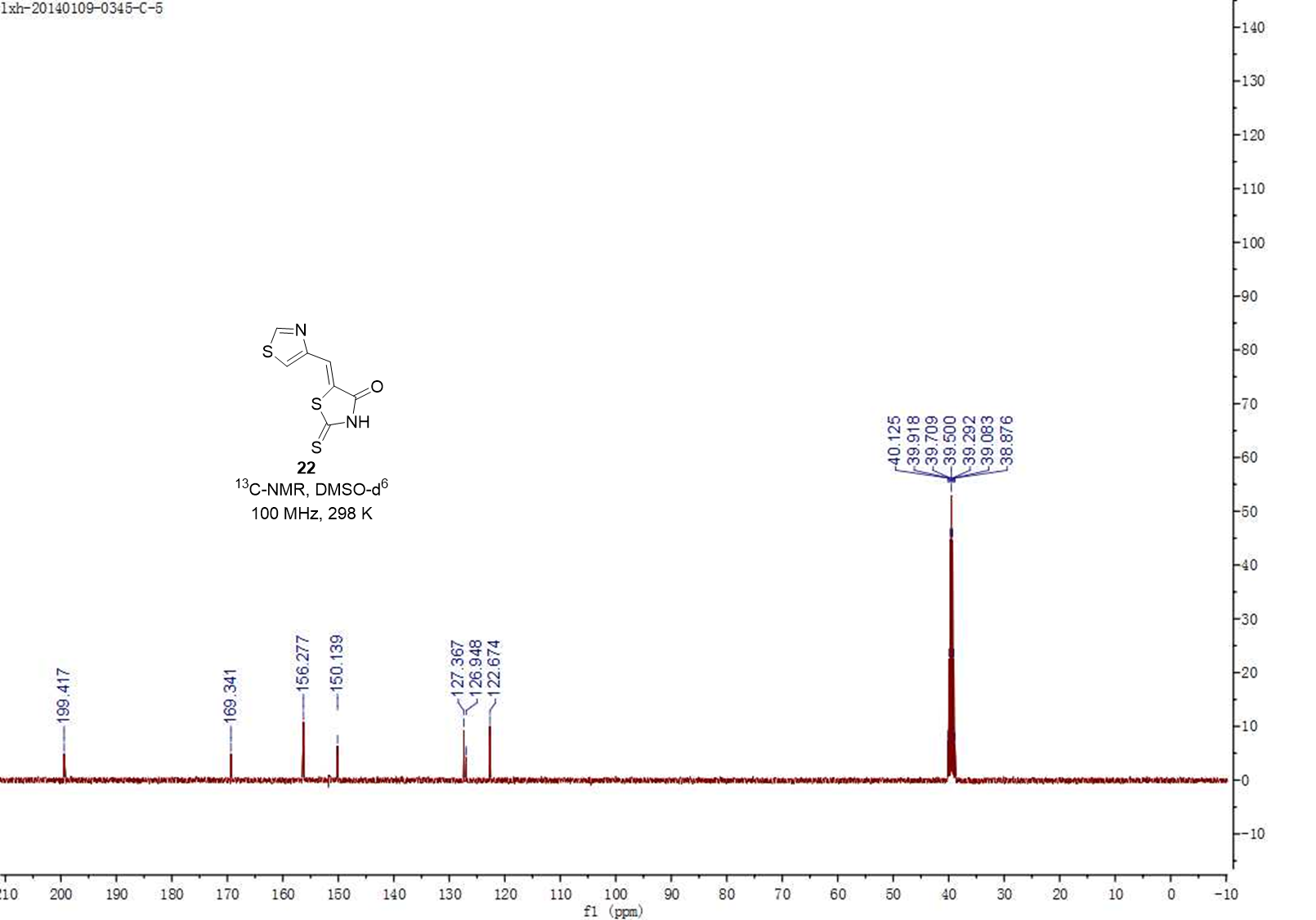

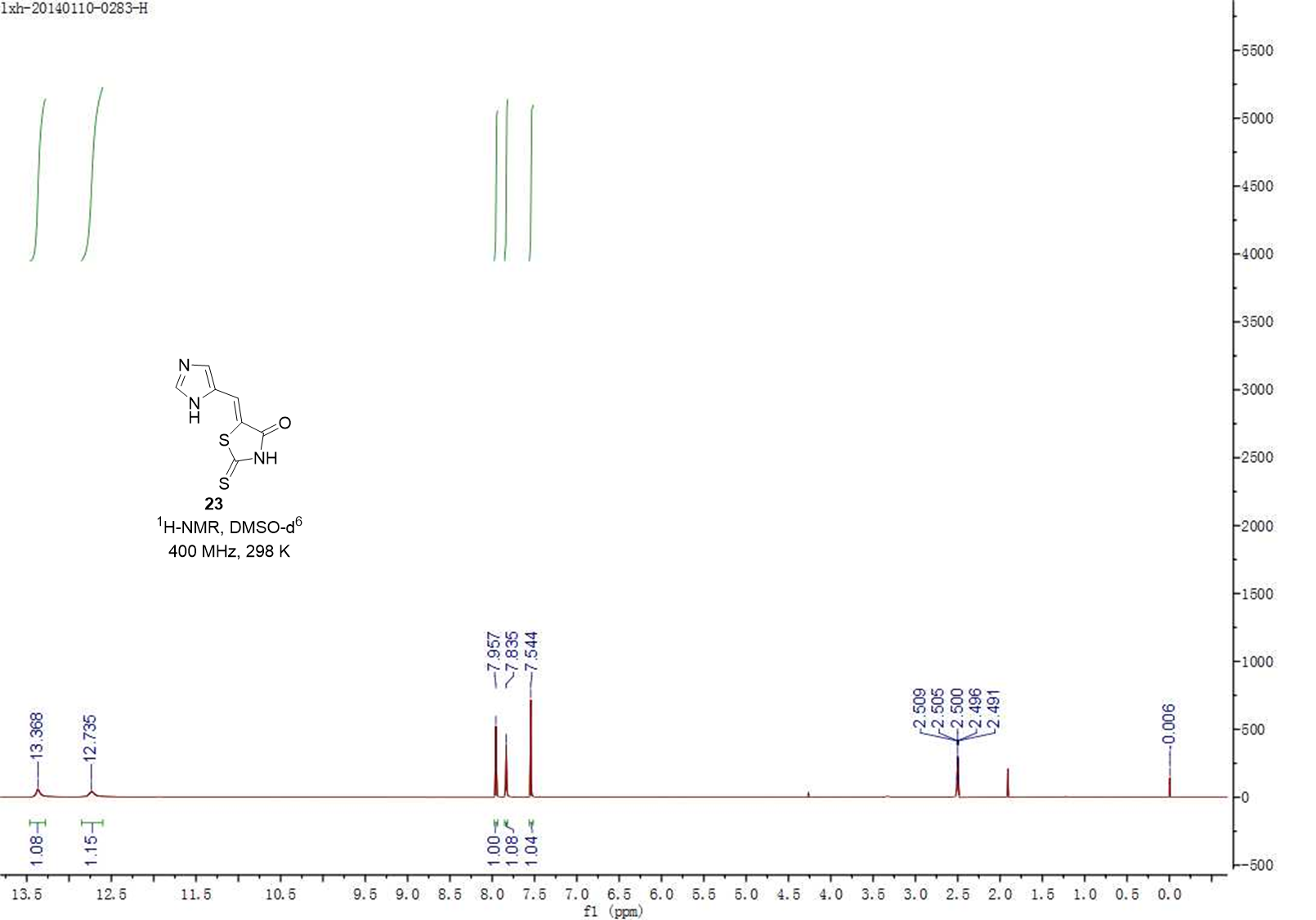

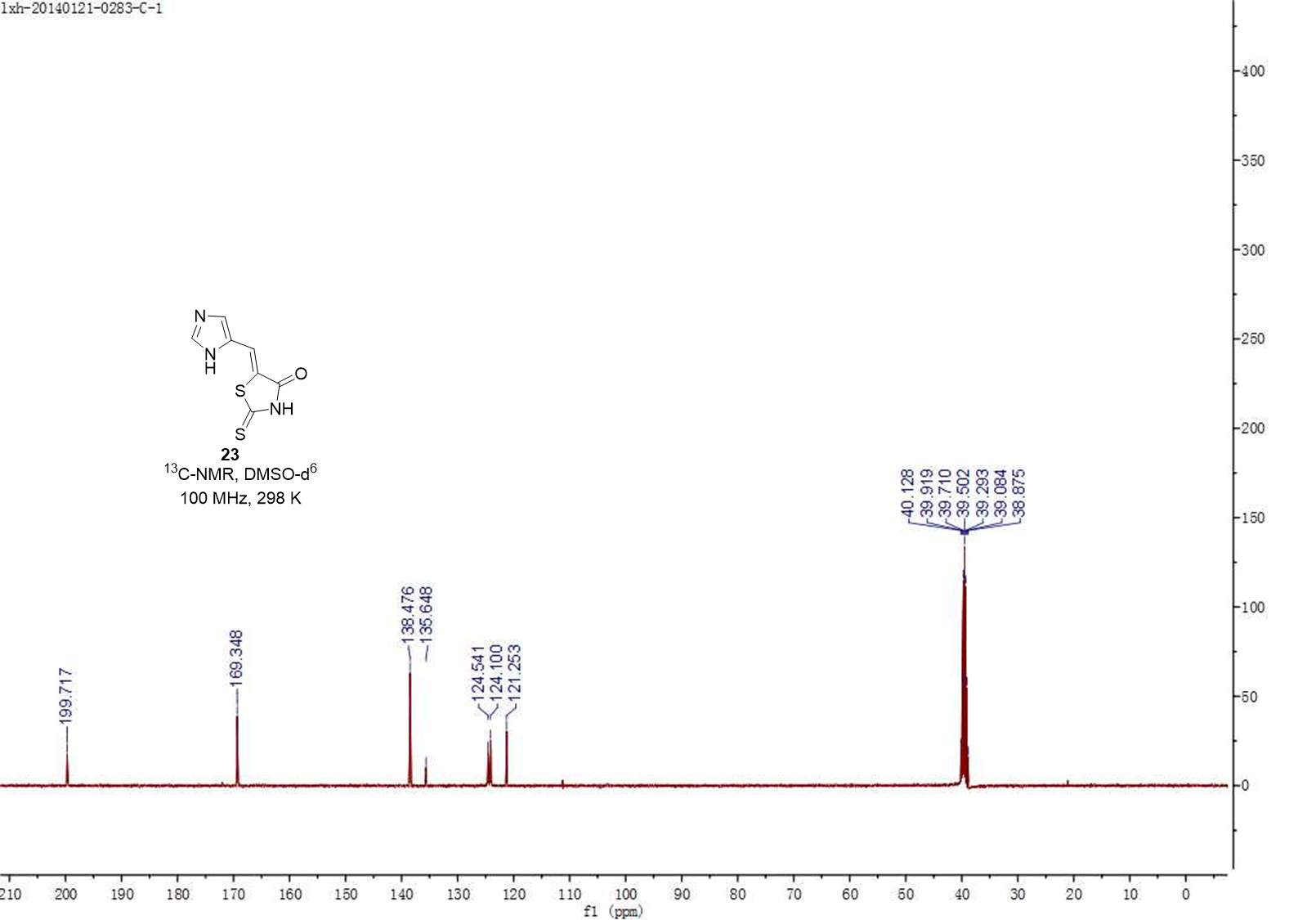

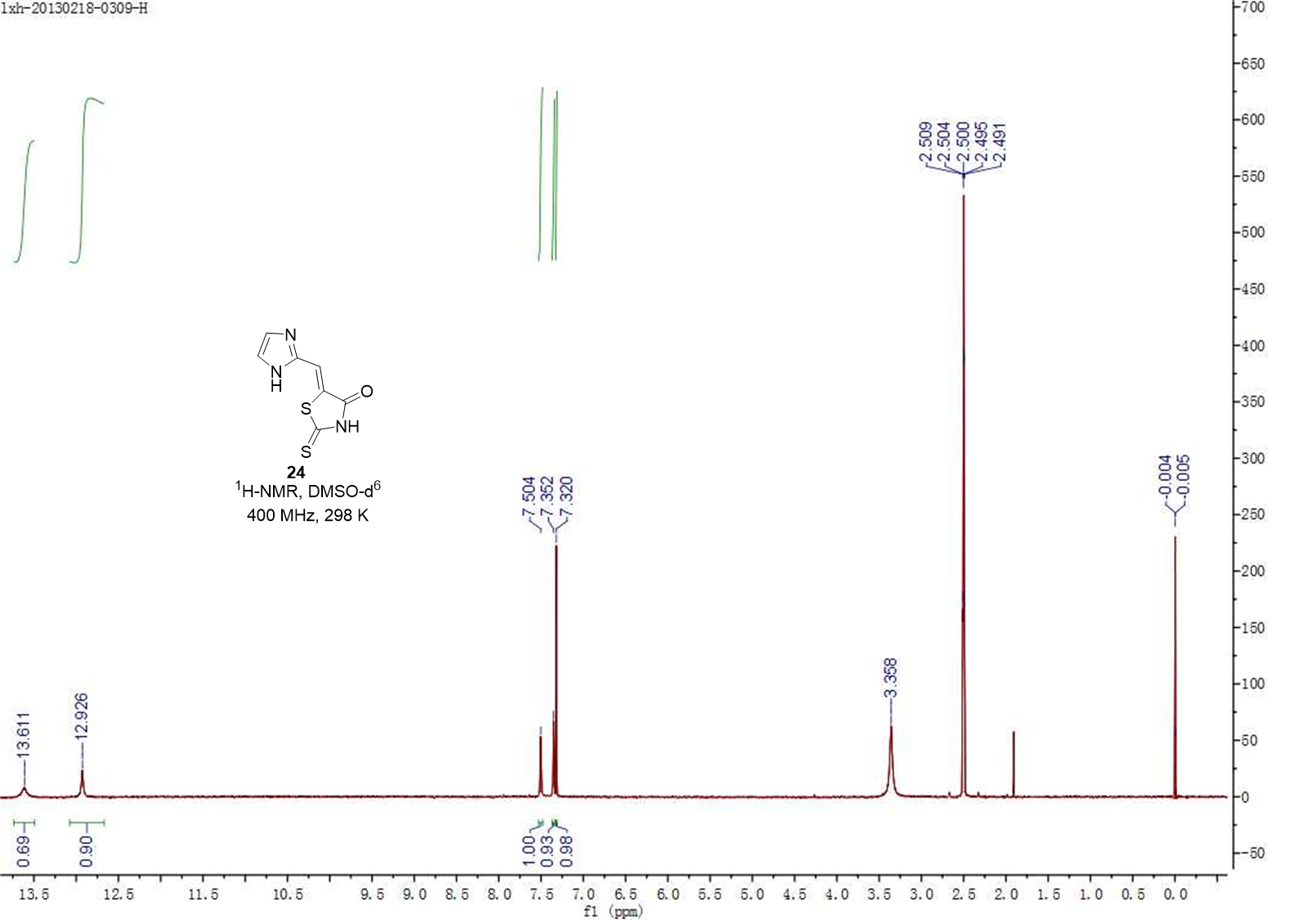

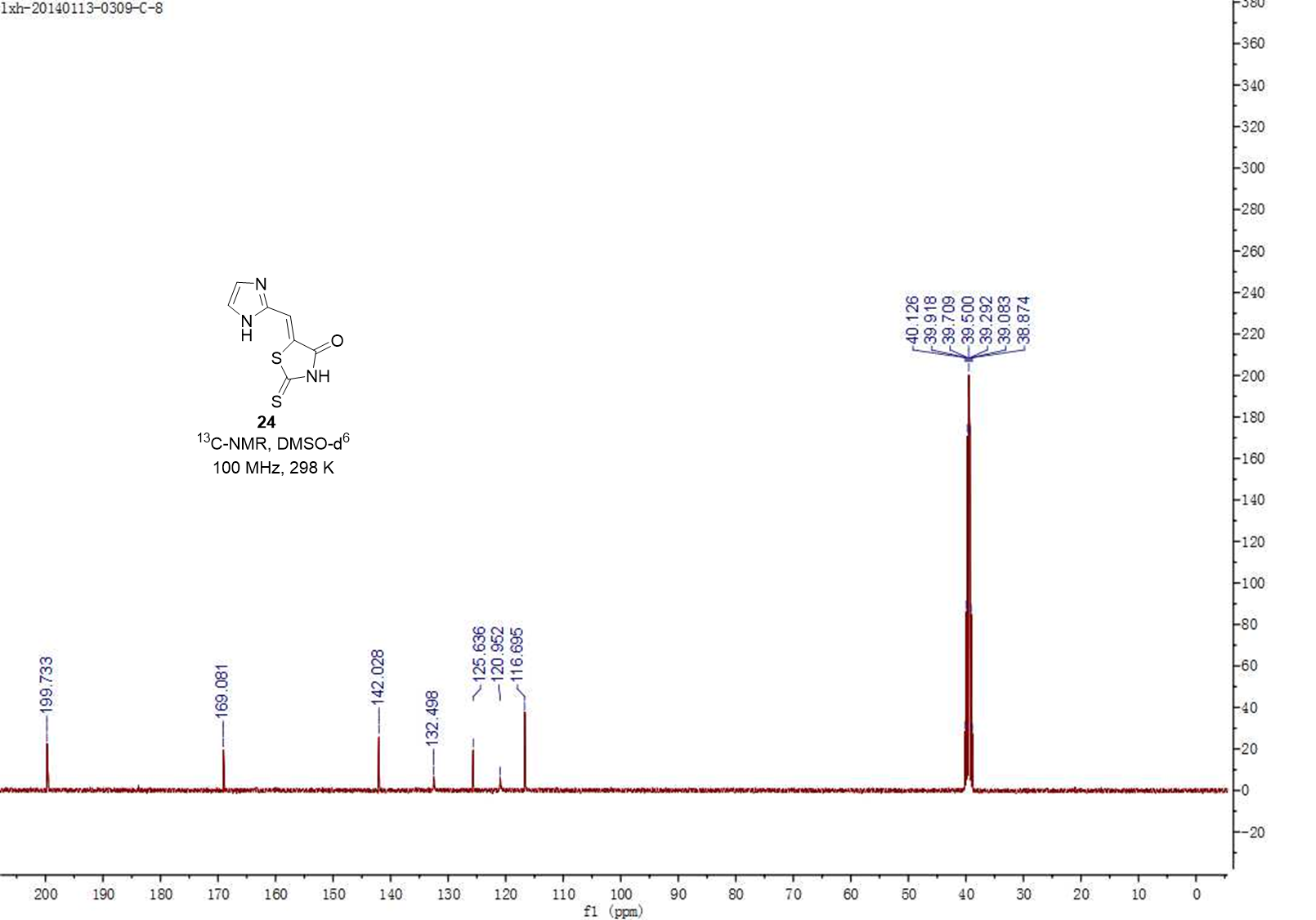

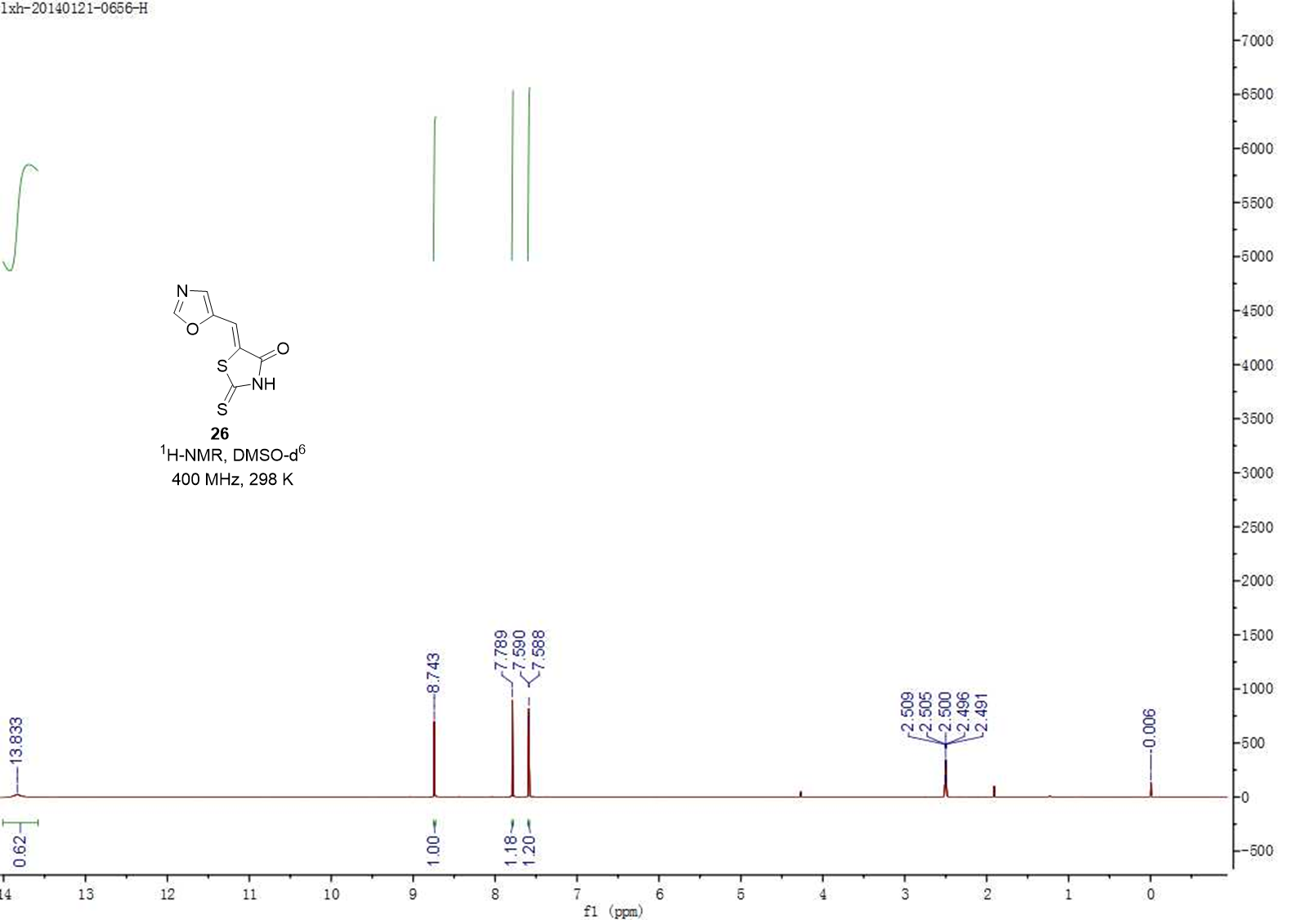

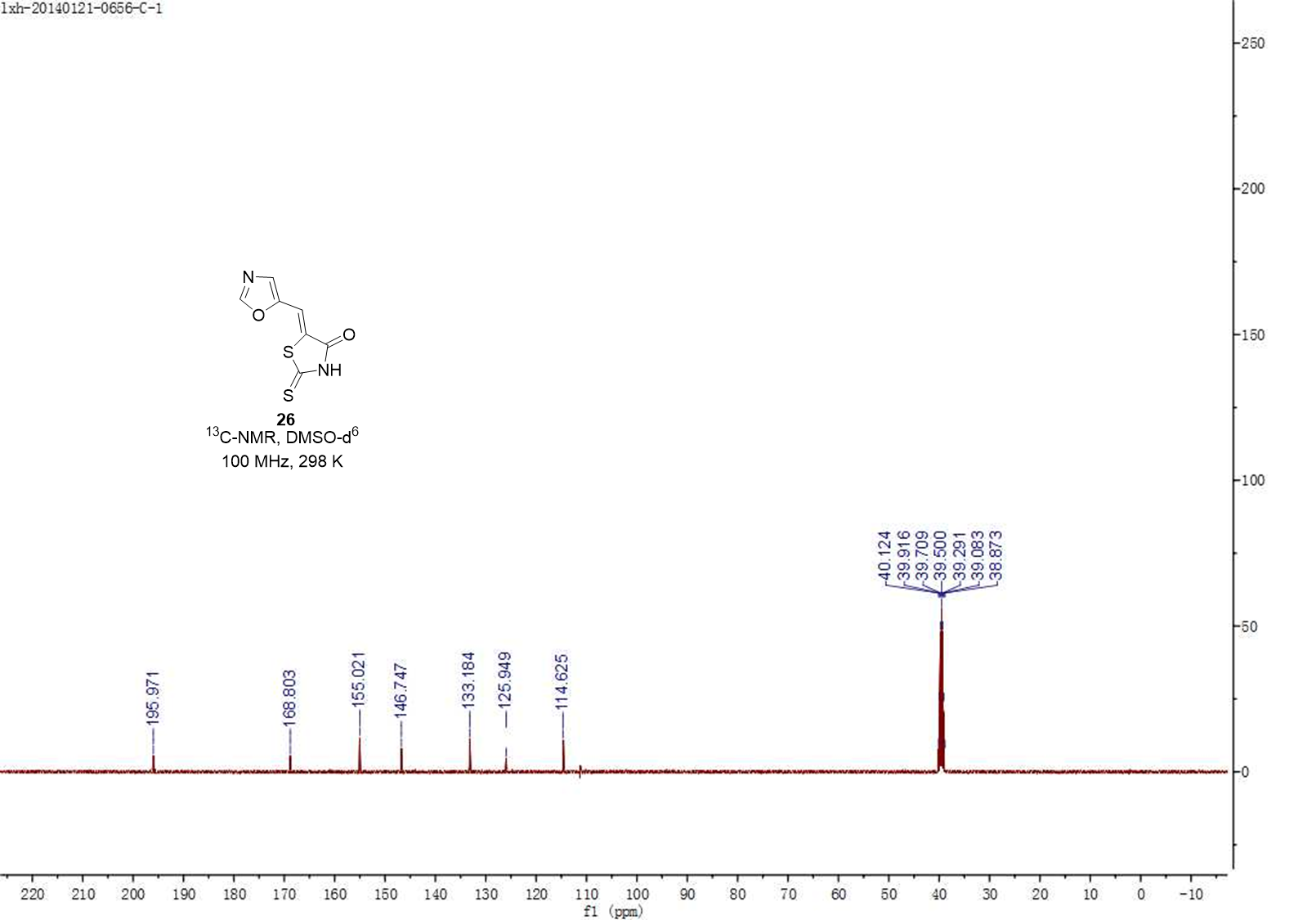

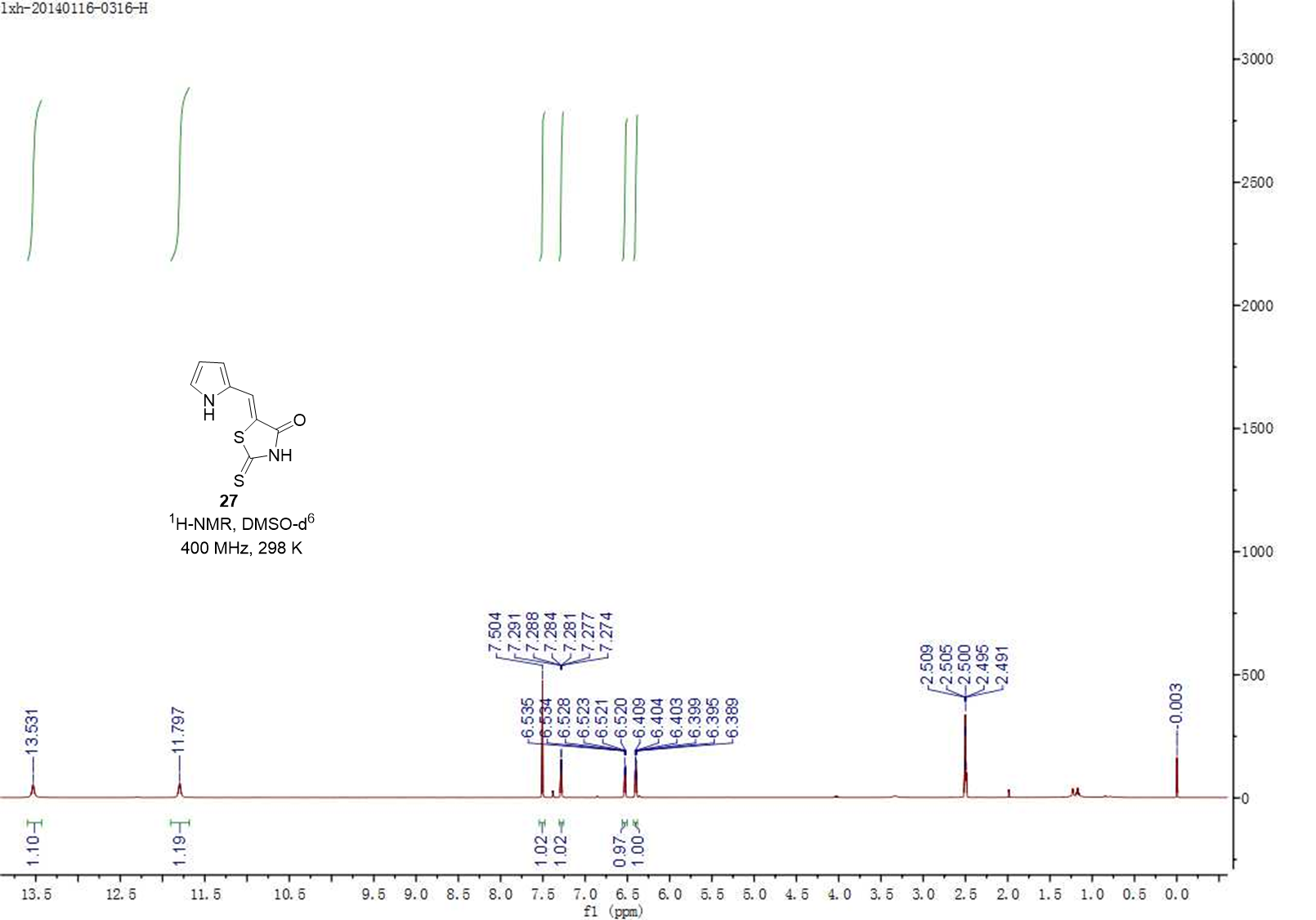

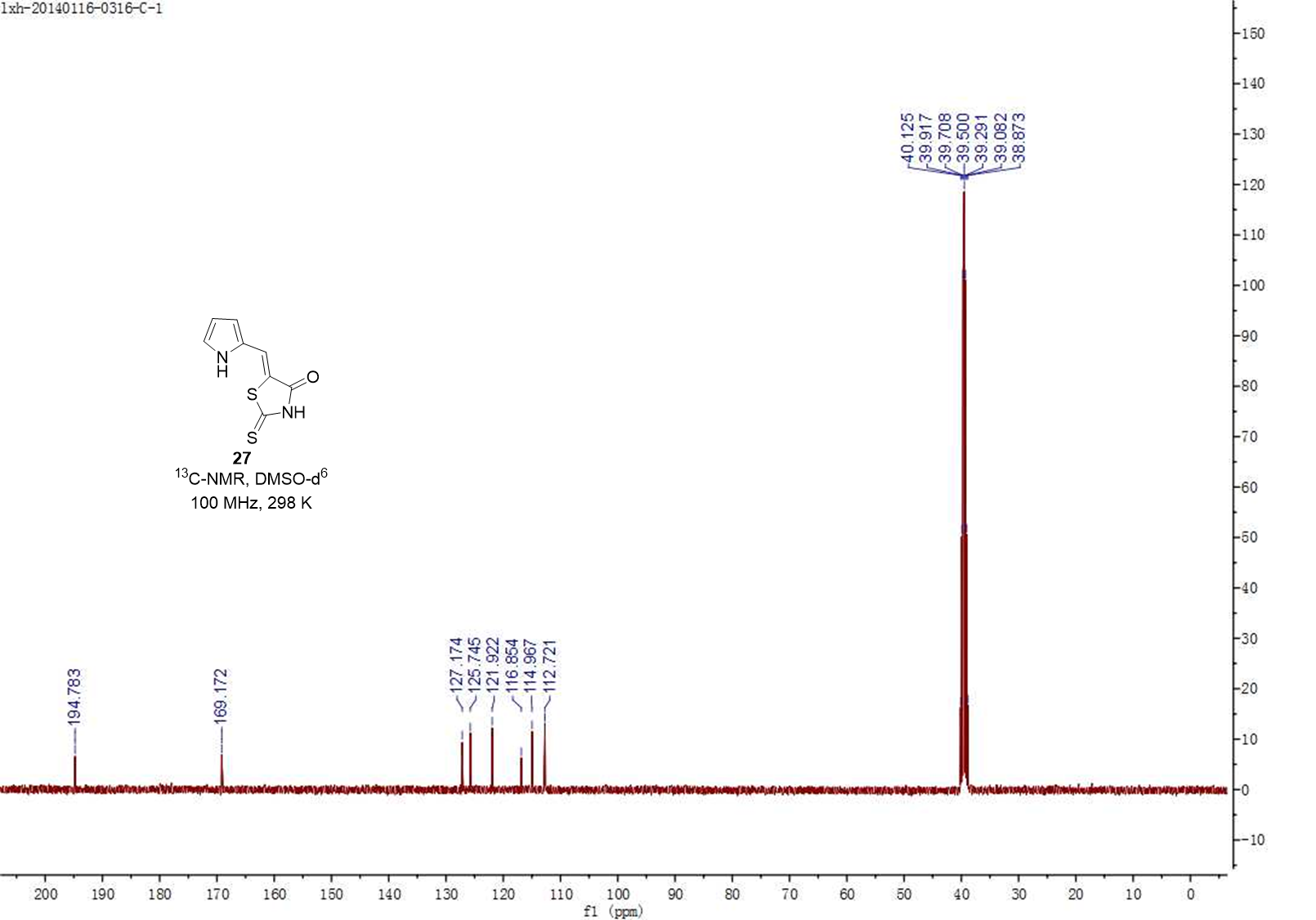

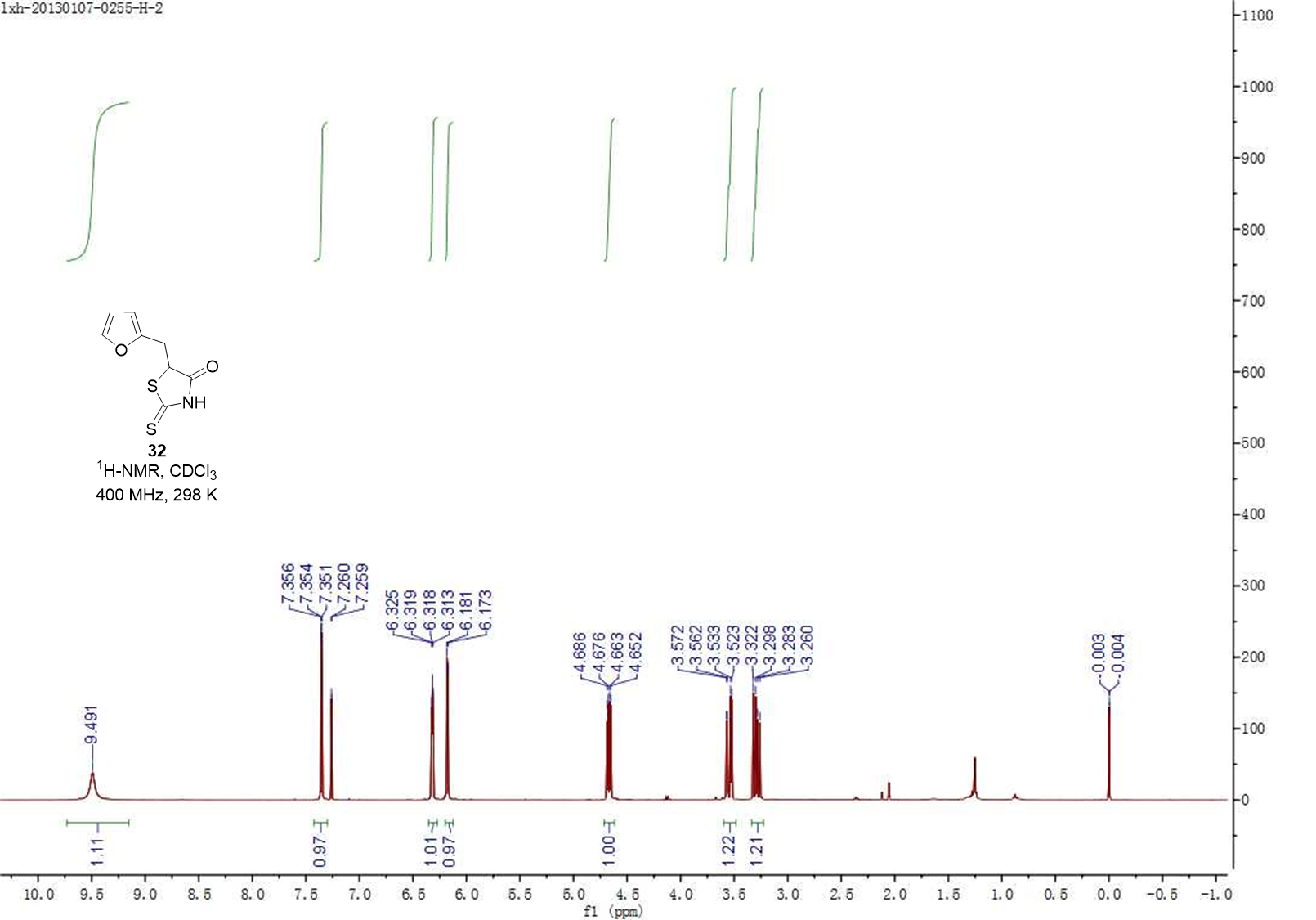

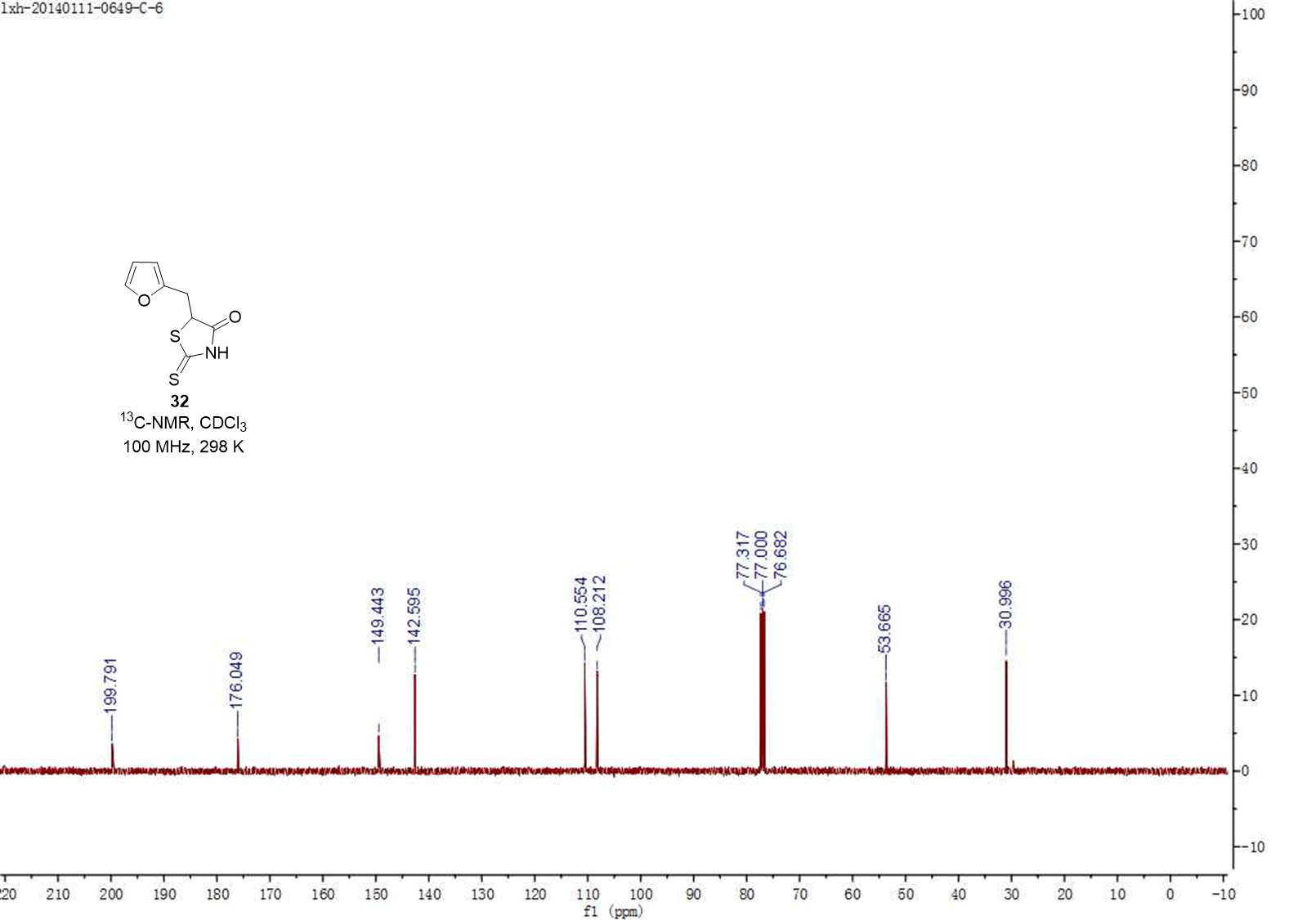

Supplement: Supplementary file 1 [file DataSheet_1.docx]
